# Synthesis, X-ray crystallography and antimicrobial activity of 2-cyanoguanidinophenytoin

**DOI:** 10.1038/s41598-023-45533-1

**Published:** 2023-11-09

**Authors:** Ahmed F. Mabied, Amr H. Moustafa, Antar A. Abdelhamid, Taha M. Tiama, Amer A. Amer

**Affiliations:** 1 X-Ray Crystallography Lab., Solid State Physics Department, National Research Centre, Dokki, 12622 Giza Egypt; 2 Department of Chemistry, Faculty of Science, Sohag University, Sohag, 82524 Egypt; 3 Faculty of Science, King Salman International University, Ras Sudr, Sinai, 46612 Egypt; 4 Department of Basic Sciences, October High Institute of Engineering & Technology - OHI, 6th of October City, Giza Egypt

**Keywords:** Drug discovery, Chemistry, Materials science

## Abstract

The optimized synthesis of [5-oxo-4,4-diphenylimidazolidin-2-ylidene]cyanamide, which is known as 2-cyanoguanidinophenytoin (CNG-DPH) (3), and (imidazo[4,5-d]imidazole-2,5-diylidine)dicyanamide (4) has been reported in the present work. Furthermore, new Mannich bases derived from CNG-DPH were synthesized via its reaction with formaldehyde and using the corresponding amines, piperidine (base 5), and morpholine (base 6). Also, the antimicrobial activity and X-ray crystal structures for CNG-DPH and their Mannich bases were studied. The bases **3** and **6** crystallized in a monoclinic system; the crystal structure of **3** containing four molecules in the unit cell with a *P*2_1_/*c* space group. The unit cell of **6** has eight molecules with a *C*2/*c* space group. The inter and intra hydrogen bond contacts packed and stabilized both of the structures. The morpholine ring of base **6** demonstrated a distinctive chair configuration. Mannich bases **5** and **6** showed promising antimicrobial effects. base **4** has a greater percentage for in vitro cytotoxicity (IC_50_) against normal cells, whereas **3** has the lowest ratio.

## Introduction

**Figure 1:**
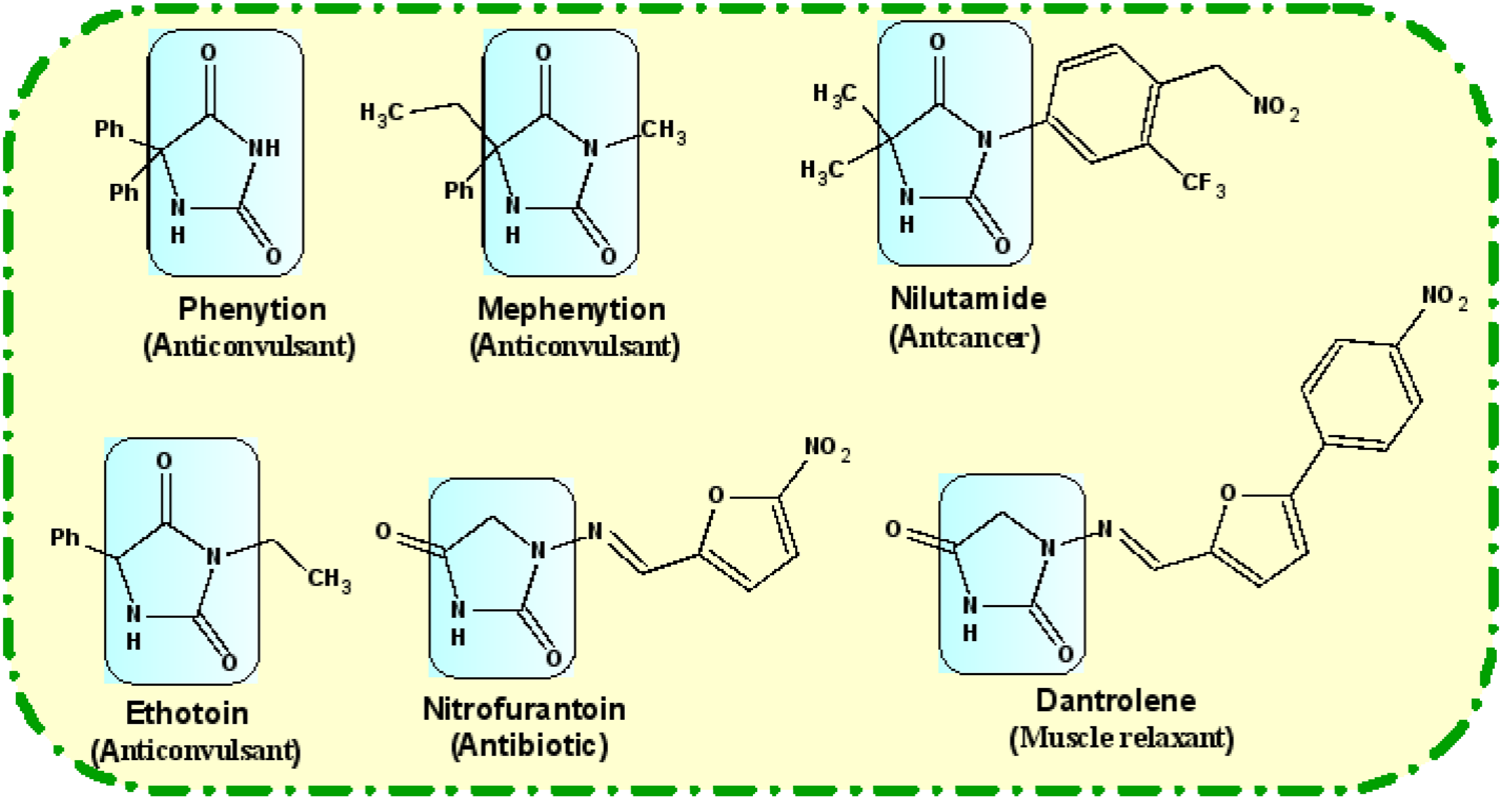
Examples of hydantoin marketing drugs.

Derivatives of hydantoin possess a significant biological activities, such as antitumor^1^, antiarrhytmics^2^ and anticonvulsants^3,4^, antiviral^5^, antineoplastic agents^6^. Nowadays, derivatives of hydantoin have found in many marketing drugs for examples: phenytoin is well known drug for treatment of epilepsy^7–9^; ethotoin and mephenytoin have anticonvulsant properties^10–12^; nilutamide is used for the treatment of prostate cancer^13,14^; nitrofurantoin is known as antibiotic^15,16^ and dantrolene is used as a muscle relaxant^17,18^ (Fig. 1). Substituted hydantoins are also used as intermediates for the synthesis of amino acids^19,20^.

**Scheme 1:**
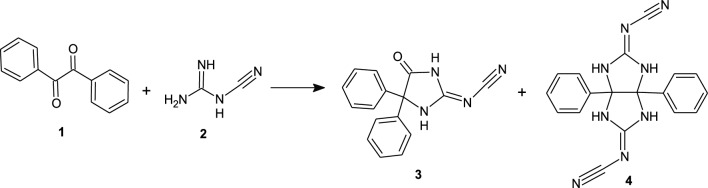
Reaction of benzil **1** with cyanoguanide **2**.

The target compound CNG-DPH (**3**) has been previously synthesized in low yield by refluxing benzil with cyanoguanidine in the presence of KOH as basic catalyst^21,22^. In continuation of our previous works in use cyanoguanidine for synthesis several of heterocyclic compounds^23–29^ and due to the biological activities of **3**^30^, we were conducted in order to improve the yield of **3** out of dramatically diminished the side product (3a,6a-diphenyltetrahydroimidazo[4,5-*d*]imidazole-2,5(1*H*,3*H*)-diylidene)dicyanamide **(4**) and consequently the yield of **3** substantially increased (Scheme 1).

Mannich bases have been reported to have potential antimicrobial activity and are effective against gram-negative microorganisms^31–35^. It has been demonstrated that the *N*-Mannich bases break down quickly in aqueous solution, forming stoichiometric amounts of formaldehyde, amine, and parent molecule, which make it useful for medical applications^36,37^. However, further evaluation of the bioactivity of such bases using different assessments methods and concentrations is still needed for more important pharmacological applications.

The present work reports the optimized synthesis, antimicrobial activity and X-ray crystal structures for **3** and their Mannich bases.

## Results and discussion

### Chemistry

The synthetic approach to CNG-DPH **3** and (imidazo[4,5-*d*]imidazole-2,5-diylidine)dicyanamide **4** is shown in Scheme 1. The initial experiments demonstrated that, the reaction of benzil **1** with an equivalent amount of cyanoguanidine **2** under refluxing in an alcoholic potassium hydroxide (0.1 equiv.) as basic catalyst for 2 h (mentioned by TLC) afforded undesired product **4** (18% yield as insoluble precipitate from hot ethanol) and the desired product **3** (57% yield), which is easily separated from filtrate after neutralization into pH ~ 2 using hydrochloric acid (Table 1, entry 1). Therefore, the reaction was optimized and examined to improve the yield of **3** and reduce the yield of compound **4** by changing various reaction conditions including the base concentration and the equivalent amount of cyanoguanidine. All results are summarized in Table 1.

**Table 1:** Optimization of the synthesis of CNG-DPH **3** and (imidazo[4,5-*d*]imidazole-2,5-diylidine)dicyanamide **4**.

Entry	Base (n equivalent)	Solvent	Cyanoguanidine (n equivalent)	Time (h)	Yield%	
					**3**	**4**
1	KOH (1)	EtOH	1.0	1	57	18
2^a^	KOH(0.5)	EtOH	2.5	4	Undetected^b^	52
3	KOH (2.5)	EtOH	1.0	1	63	15
4	KOH (4)	EtOH	2.5	1	52	24
5^a^	KOH (4)	EtOH	1.0	1	65	Undetected^b^
6	NaOH (4)	EtOH	1.0	1	60	10
7	MeONa (2.5)	MeOH	1.0	1	67	12
8	EtONa (2.5)	EtOH	1.0	1	72	8
9^c^	**EtONa (4)**	**EtOH**	**1.0**	**1**	**80**	**5**
10	EtONa (5)	EtOH	1.0	1	78	5

^a^Reported method^21^.

^b^Formed as reported^21^ but their yields are not detected.

^c^Optimized condition.

Significant values are in bold.

The effect of the base on the reaction was screened with various molarities using different strong bases. While, using organic bases is not effective in reactions of cyanoguanidine^24^. The screening results indicated that sodium ethoxide and sodium methoxide are the most effective bases for this reaction (Table 1, entries 7–10). By increasing the base molarity to 4 mol equivalent, the yield of desired product **3** was improved to 80% and the yield of undesired product **4** was reduced to 5% (Table 1, entry 9), while by increasing number of moles more than 4 mol equivalent there is no improvement for the reaction yield (Table 1, entry 10). Finally, optimization of the molarity of cyanoguanidine required was screened and it was found that by increasing the molarity of cyanoguanidine (2.5 mol equivalent) increase the yield of undesired product **4** (Table 1, entries 2 and 4).

**Scheme 2:**
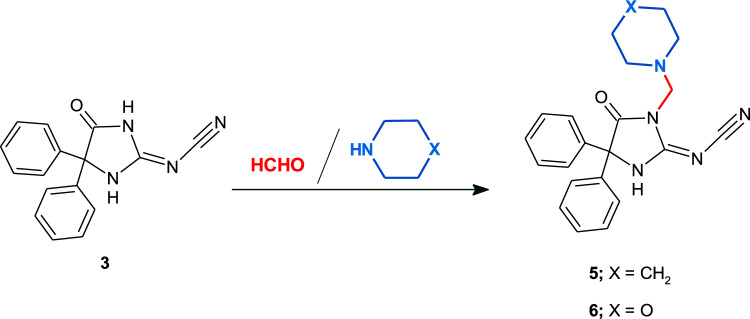
Synthesis of Mannich base **5** and **6**.

Mannich bases **5** and **6** derived from **3** were synthesized in a simple conventional manner, by stirring compound **3** and formaldehyde under refluxing ethanol for 15 min and then an equivalent mole of appropriate secondary amine; piperidine and/or morpholine was added and stirred for about 2 h. Then, the reaction mixture was cooled and the formed crystal **5** and/or **6**, respectively was filtered off and used without further purification (Scheme 2).

The chemical structures of all synthesized compounds **3–6** were confirming based on their spectral (IR, ^1^H, ^13^C, Dept-135 NMR) and elemental analysis data. For example, the IR spectrum of newly Mannich base** 6** showed an absorption band at 3114 cm^−1^ due to N–H group, 3023 cm^−1^ attributed to aromatic C–H. In addition, it showed three absorption bands at 2949, 2869, 2819 cm^−1^ characteristic for aliphatic C–H beside an absorption band at 2188 cm^−1^ due to C≡N group, which is agree with =N–≡N group^24,25,38,39^ and other absorption band at 1763 cm^−1^ attributed to C=O group. Its ^1^H NMR spectrum showed the presence of broad singlet signal at *δ* 11.23 ppm due to NH proton; it exhibited multiple signals at *δ* 7.38–7.47 ppm characteristic of ten aromatic protons; and three singlet signals in aliphatic region at *δ* 2.49, 3.51 and 4.50 ppm characteristic of CH_2_–N, CH_2_–O and N–CH_2_–N protons, respectively.

**Figure 2:**
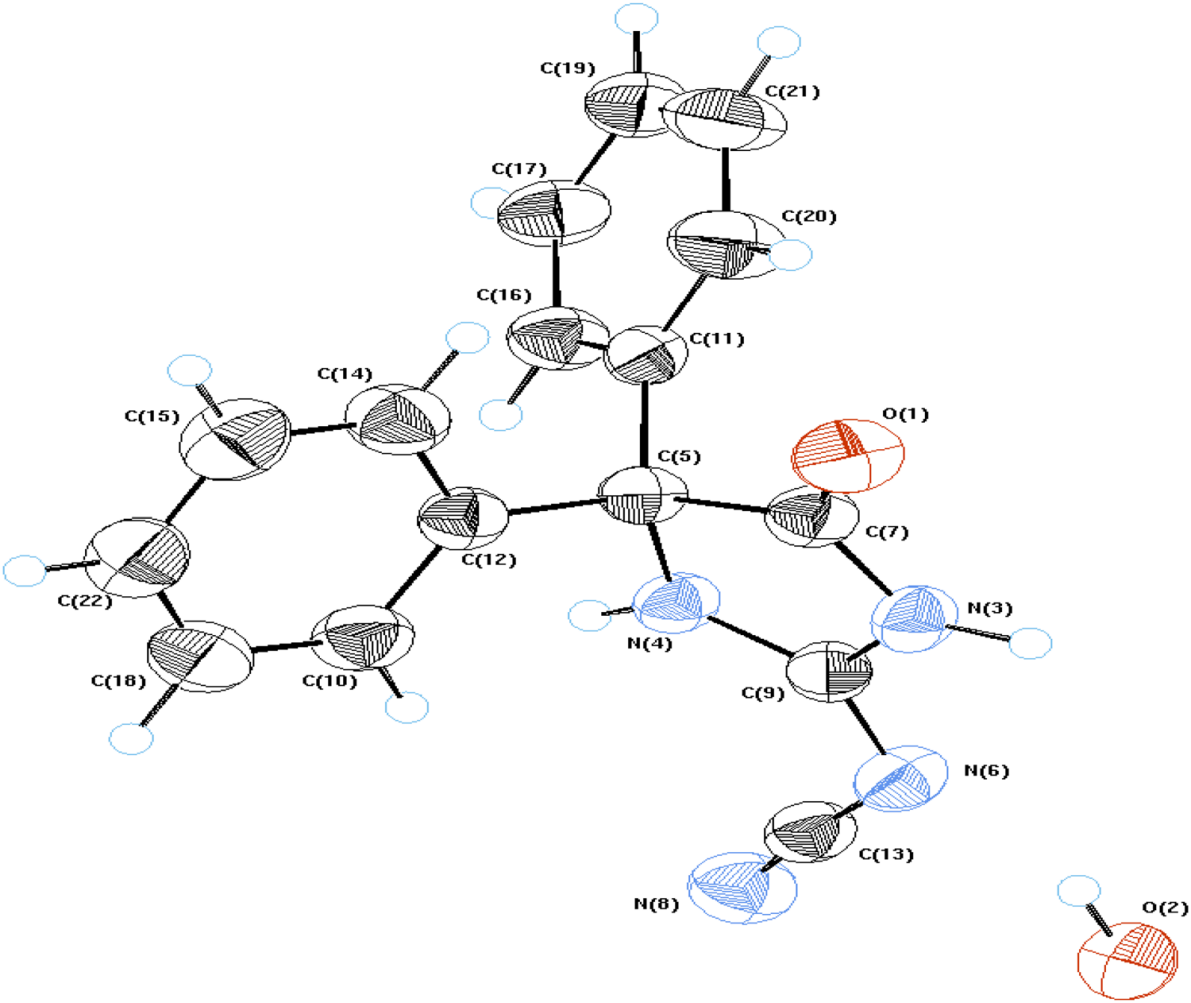
An ORTEP view of **3** with atom-numbering. Displacement ellipsoids are drawn at the 50% probability level and H atoms are shown as small spheres of arbitrary radii.

**Figure 3:**
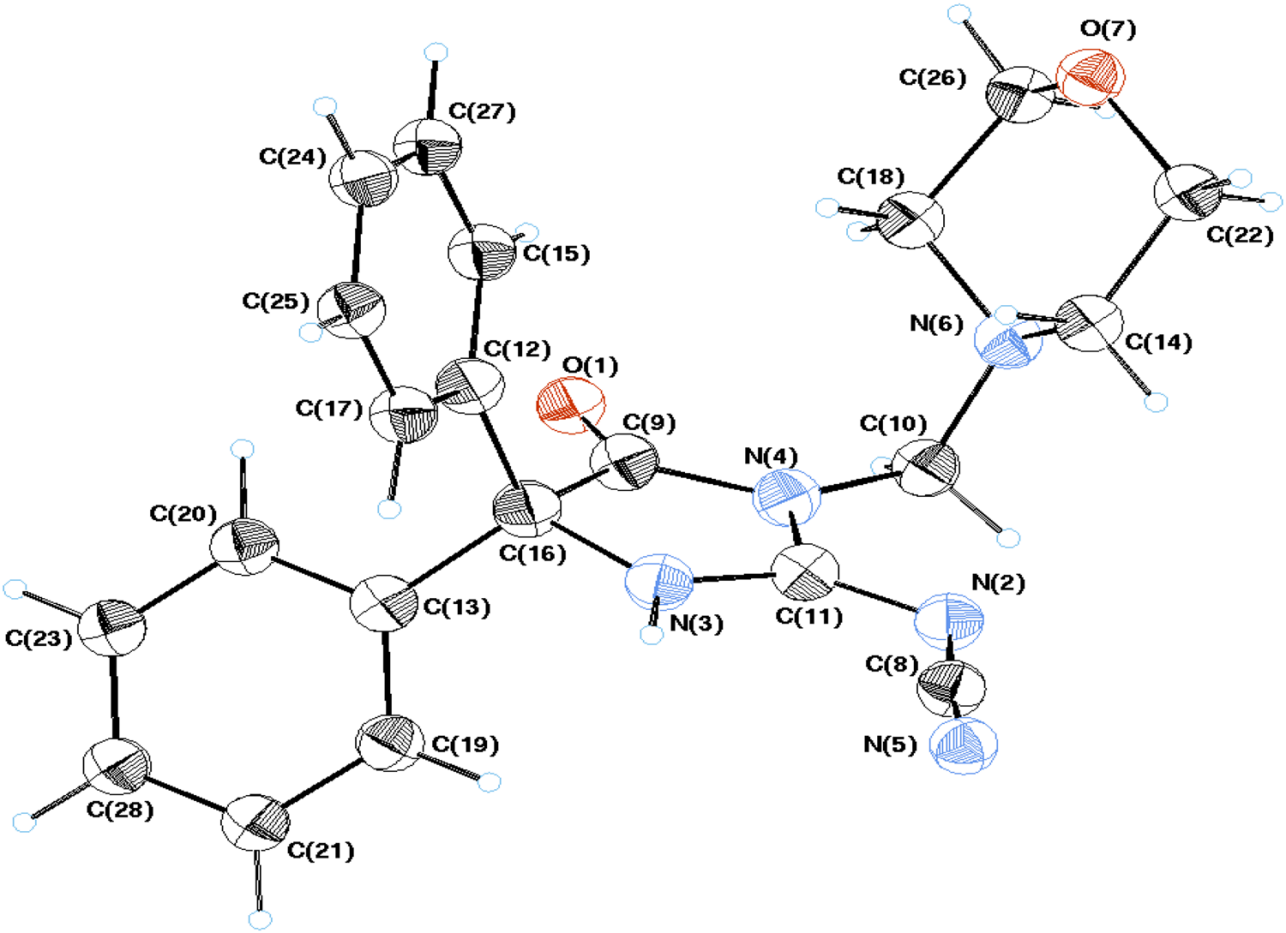
An ORTEP view of Mannich base **6** with atom-numbering. Displacement ellipsoids are drawn at the 50% probability level and H atoms are shown as small spheres of arbitrary radii.

^13^C NMR spectrum of** 6** showed six signals at *δ* 174.7, 161.1, 138.6, 129.1, 127.4 and 115.1 ppm, which are assigned to carbons of carbonyl group, aromatic and nitrile group, respectively, while aliphatic carbons of *sp*^3^ C-4, NCH_2_N, OCH_2_ and NCH_2_ are characterized by signals at *δ* 71.9, 66.5, 62.3, 51.1 ppm, respectively. Its dept-135 showed the disappearance of signal at *δ* 71.9 ppm which characteristic of C-4 and exchangeable signals attributed to methylene groups. In addition, X-ray crystallography of **3** and** 6** are shown at Figs. 2 and 3 (see “X-ray crystallography” section).

## X-ray crystallography

Figures 2 and 3 show molecular geometry of **3** and **6**, from an Ortep perspective. The compounds crystallized in the monoclinic system possessing four molecules in the unit cell and *P*2_1_*/c* of **3** and eight molecules with *C*2*/c* space group for **6**. The crystal structure of compound **3** is consistent with the same published structure^40^. The main difference between the structures of 3 and 6 is the existence of a 4-methylmorpholine moiety in the structure of 6 (Figs. 2, 3).

**Figure 4:**
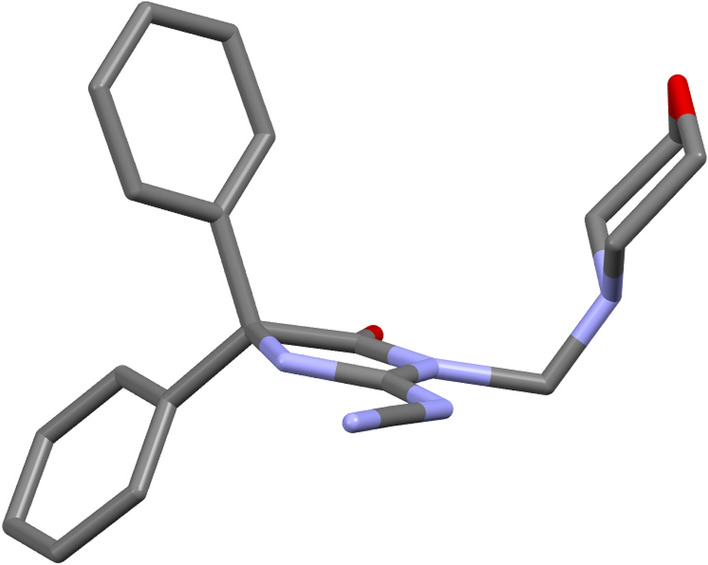
A view of compound **6** showing chair conformation in the morpholine ring.

The molecular bond geometries indicated the connectivity of the atoms and were in agreement with the reported standard bond distances^41^. Calculations of the least-squares plane passing through the consisting atoms showed general planar configurations at **3** in every ring individually. However, all of the constituent parts exist in different planes. The phenyl rings have an angle 78.8° at **3** in agreement with the published structure (78.9°)^40^ and 88.12° for **6** between the normal of their planes. The imidazole ring (N3/C11/N4/C9) in **6** showed non planar character, with maximum deviation from the plane at C9 (0.034 Å), and torsion angle N3/C11/N4/C9 (2.35°). Puckering investigations at 6-membered morpholine ring (O7/C22/C14/N6/C18/C26) of 6 indicated an obvious chair conformation (Fig. 4)^42^.

**Figure 5:**
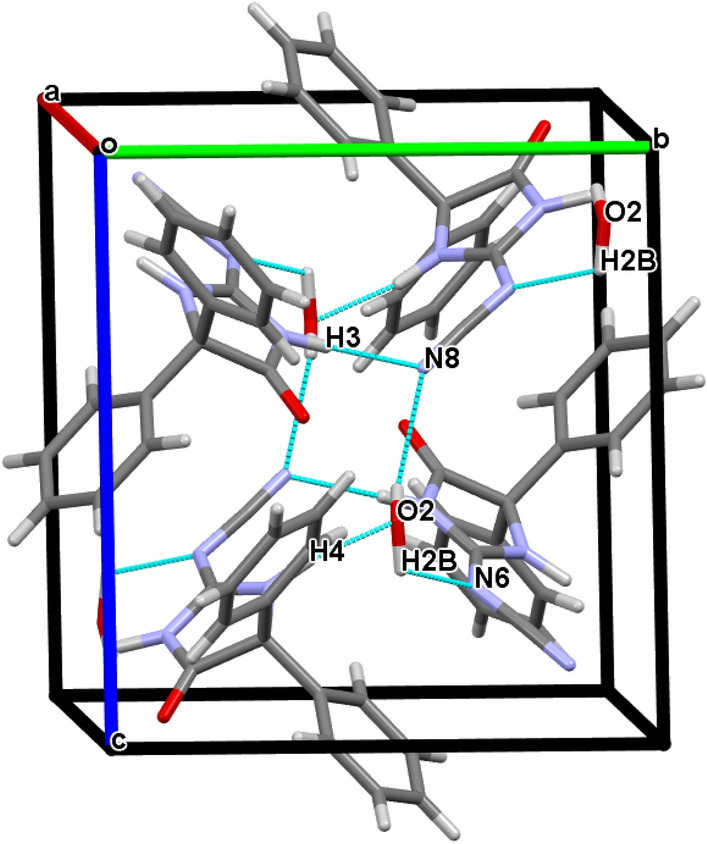
A view of packing diagram for compound **3**. Hydrogen bond contacts with dashed blue lines.

**Figure 6:**
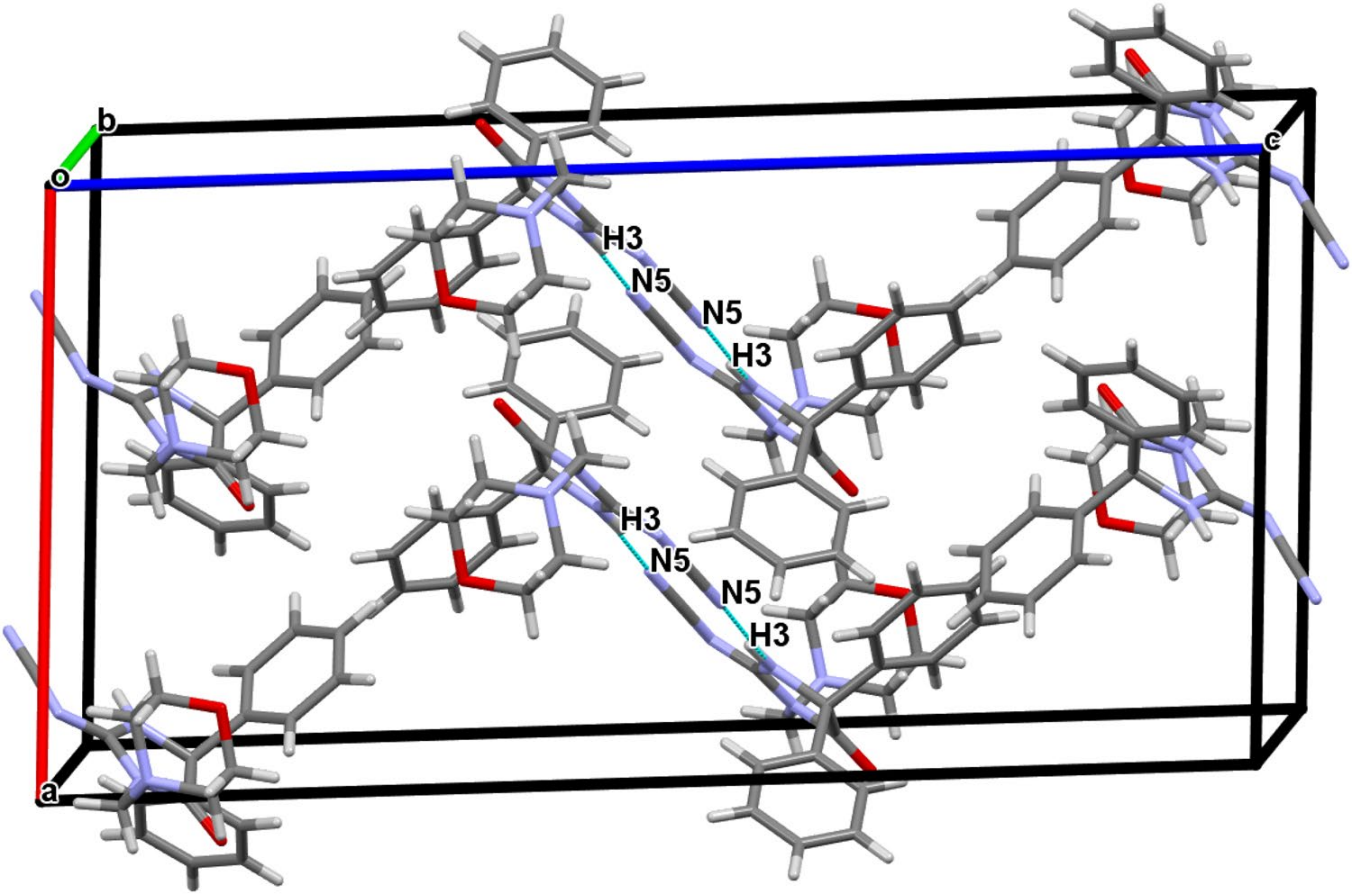
A view of packing diagram for compound **6**. Hydrogen bond contacts with dashed blue lines.

The structures are stabilized by the inter and intra molecular networks of hydrogen bond contacts, conformed parallel layers. The crystal packing is further stabilized by rings interaction (Cg-J), where Cg refers to the gravity center of a ring (J), and J identifies the ring number in a structure. It was found that the 5-Membered imidazole ring (Cg1) in compound **3**, as well as the morpholine ring (Cg4) in compound **6**, have gravity centers that interact with hydrogens (Tables 2, 3). The packing diagrams and intermolecular contacts of the compounds **3** and **6** are shown in Figs. 5 and 6, respectively.

**Table 2:** Molecular contacts geometry (Å, °) of **3**.

*D*—H⋯*A*	*D*—H	H···*A*	*D*···*A*	*D*—H···*A*
O2—H2B⋯N6^i^	0.96	2.53	2.955(3)	107
N3—H3⋯N8^ii^	0.96	2.07	3.024(3)	172
N4—H4⋯O2	0.96	1.89	2.836(3)	168
C10—H10⋯N4 intra	0.96	2.44	2.820(3)	103
C20—H20⋯O1 intra	0.96	2.36	3.048(4)	128
C10—H10⋯Cg1	0.96	2.93	3.107	92.00

Symmetry codes: (i) 1 − x, − 1/2 + y, 1/2 − z; (ii) 1 − x, − y, − z.

**Table 3:** Molecular contacts geometry (Å, °) of **6**.

*D*—H⋯*A*	*D*—H	H⋯*A*	*D*⋯*A*	*D*—H⋯*A*
N3—H3⋯N5i	0.96	1.99	2.928(5)	164
C15—H15⋯O1 intra	0.96	2.49	3.146(5)	125
C19—H19⋯N3 intra	0.96	2.46	2.843(5)	104
C26—H26B⋯Cg4ii	0.96	2.90	3.832(2)	164.00

Symmetry codes: (i) 3/2 − x,1/2 − y, − z (ii) X, − 1 + Y, Z.

## Antimicrobial activity

**Figure 7:**
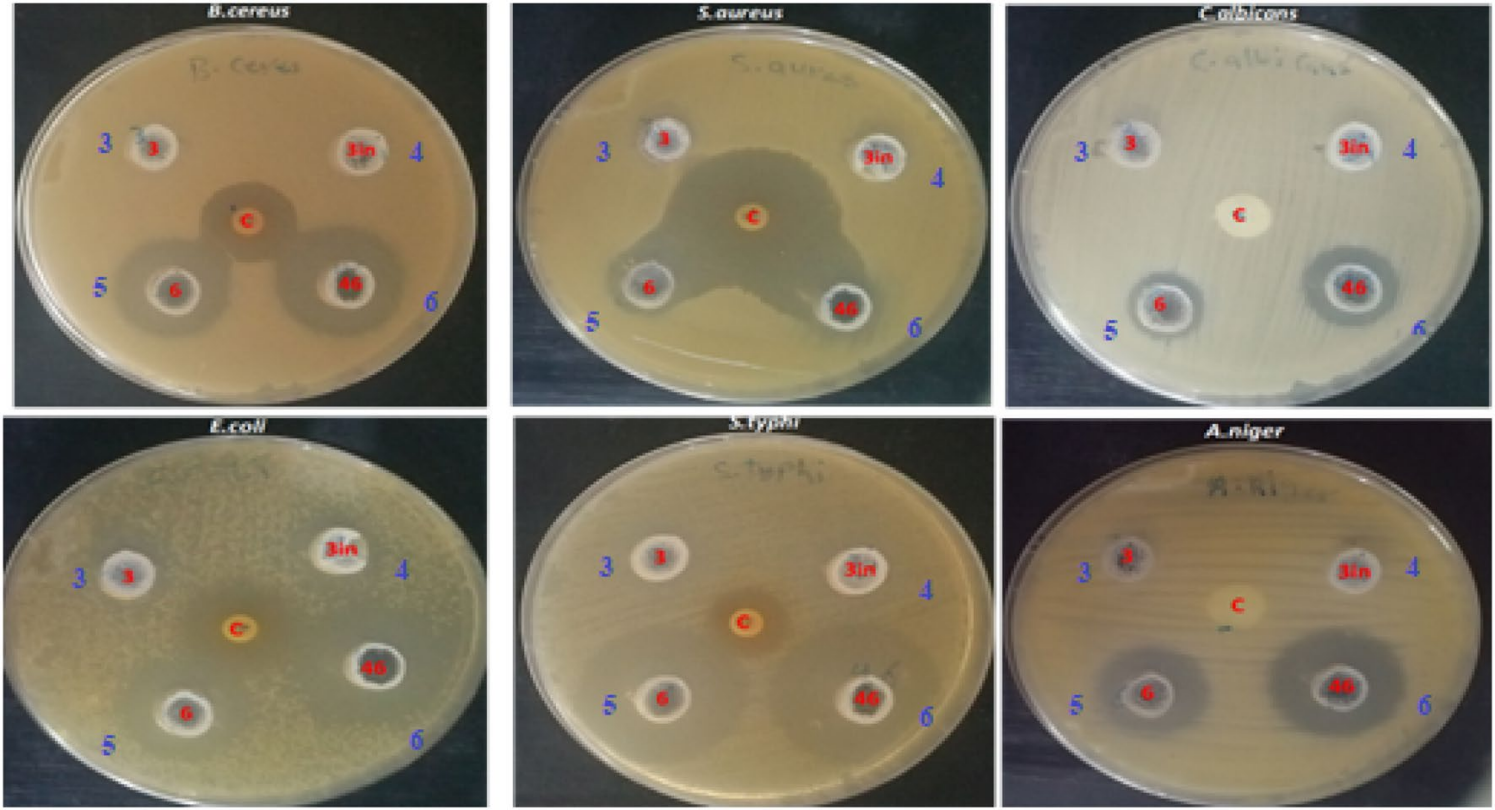
Inhibition zone diameter for compounds **3–6.**

The newly synthesized compounds were tested for their antimicrobial activity against the following microorganisms: two gram positive stains *B. cereus, S. aureus P*. and, two gram negative stains aeruginosa *E. coli*, *S. typhi*, and two fungal stains *Candida albicans* and *Aspergillus niger*. The preliminary screening of the investigated compounds was performed using Broth dilution method. The minimum inhibitory concentrations, were recorded accordingly. The antimicrobial activity of Mannich bases **5** and **6** was active against both Gram-positive, Gram-negative bacteria, *Candida albicans* and *Aspergillus niger* with different inhibition zone diameter range from 15.33 to 30.16 mm as mentioned in Table 4 and Fig. 7.

**Table 4:** Antimicrobial activity of compounds **3–6**.

Microbial strain	Inhibition zone (mm) at concentration 10 mg/ml				Rifampin/fluconazole
	**3**	**4**	**5**	**6**	
*S. aureus*	0	0	16.66 ± 0.33	15.33 ± 0.33	34 ± 0.57
*B. cereus*	0	0	20 ± 0.57	17 ± 0.57	17.33 ± 0.33
*E. coli*	13.3 ± 0.33	14.66 ± 0.66	27.33 ± 0.33	23.33 ± 0.33	22 ± 0.577
*S. typhi*	0	0	30.16 ± 0.44	29.3 ± 0.88	11.33 ± 0.33
*C. albicans*	0	0	20 ± 0.57	15 ± 0.57	0
*A. niger*	0	0	25.66 ± 0.33	22.33 ± 0.33	0

The table shows the inhibition zone (mm) of different microbial strains at a concentration of 10 mg/ml of different compounds. The inhibition zone is a measure of the antimicrobial activity of the tested compounds against the microbial strains. The results indicate that the tested compounds have varying degrees of antimicrobial activity against the different microbial strains.

Compounds **3** and **4** didn’t show antimicrobial activity except *E. coli* with minimum inhibition zone 13.3 and 14.6 mm respectively: Rifampin demonstrated an inhibition zone diameter range from 11.3 to 34 mm against tested bacteria whereas fluconazole didn’t demonstrate an inhibition zone against both *C. albicans* and* A. niger.*

It is important to note that the results of the study are limited to the specific concentrations and strains tested. Further studies are needed to determine the minimum inhibitory concentration (MIC) and the mechanism of action of the tested compounds against the different microbial strains. The results of the study may have implications for the development of new antimicrobial agents and the treatment of microbial infections.

## In vitro cytotoxicity

### Samples assessment

**Figure 8:**
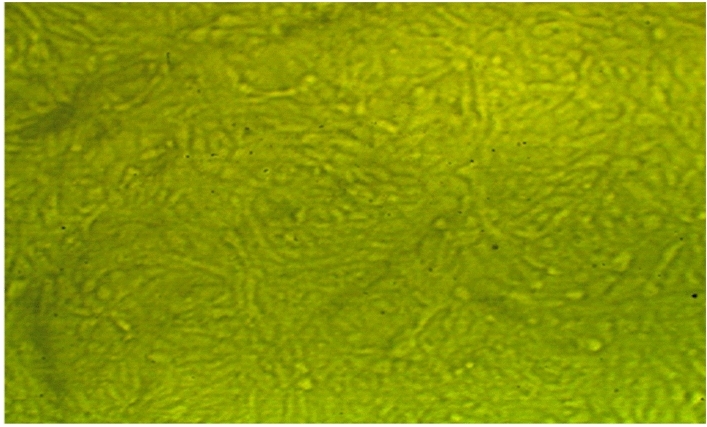
Control of Vero cell.

**Figure 9:**
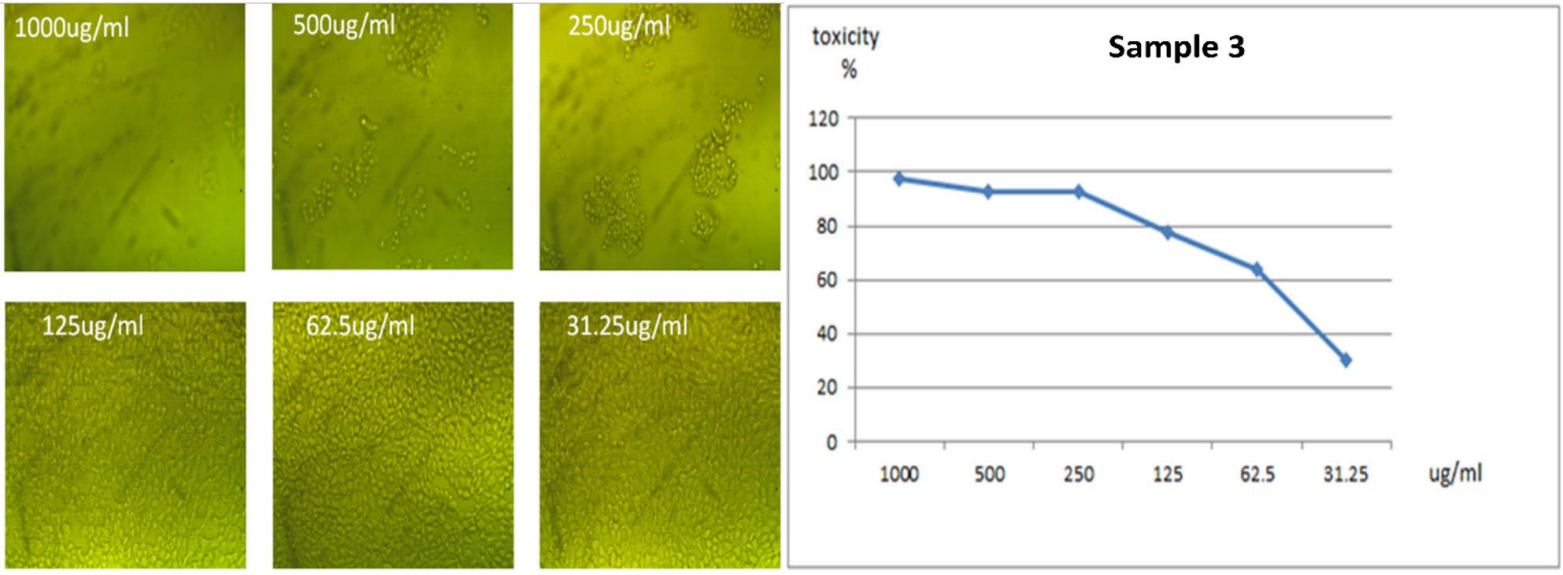
Effect of compound **3** on Vero cell at different concentration.

**Figure 10:**
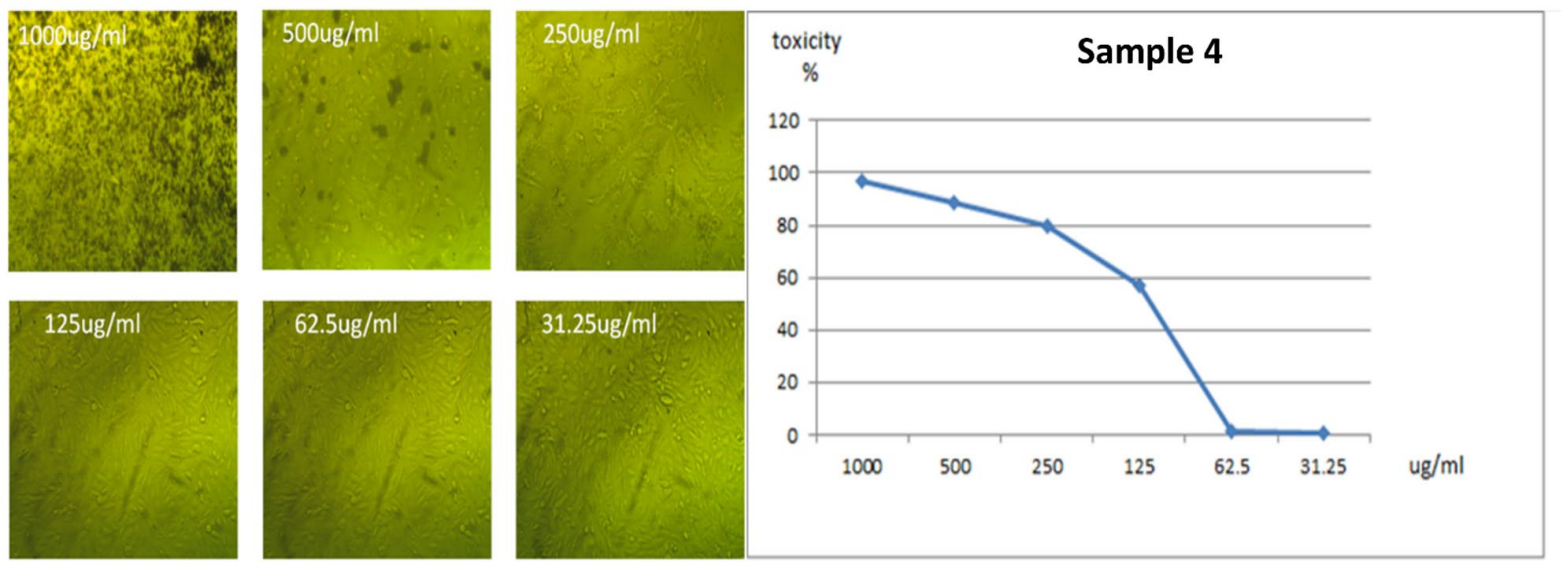
Effect of compound **4** on Vero cell at different concentration.

**Figure 11:**
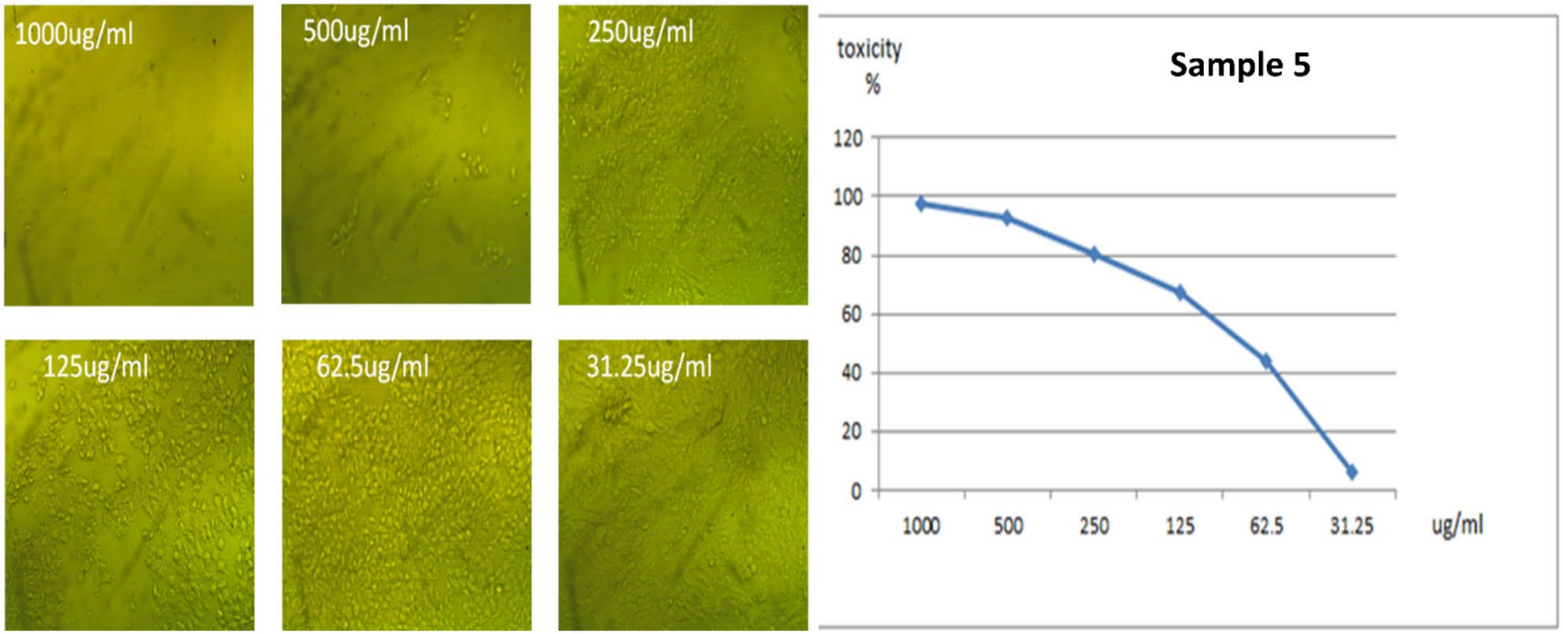
Effect of compound **5** on Vero cell at different concentration.

**Figure 12:**
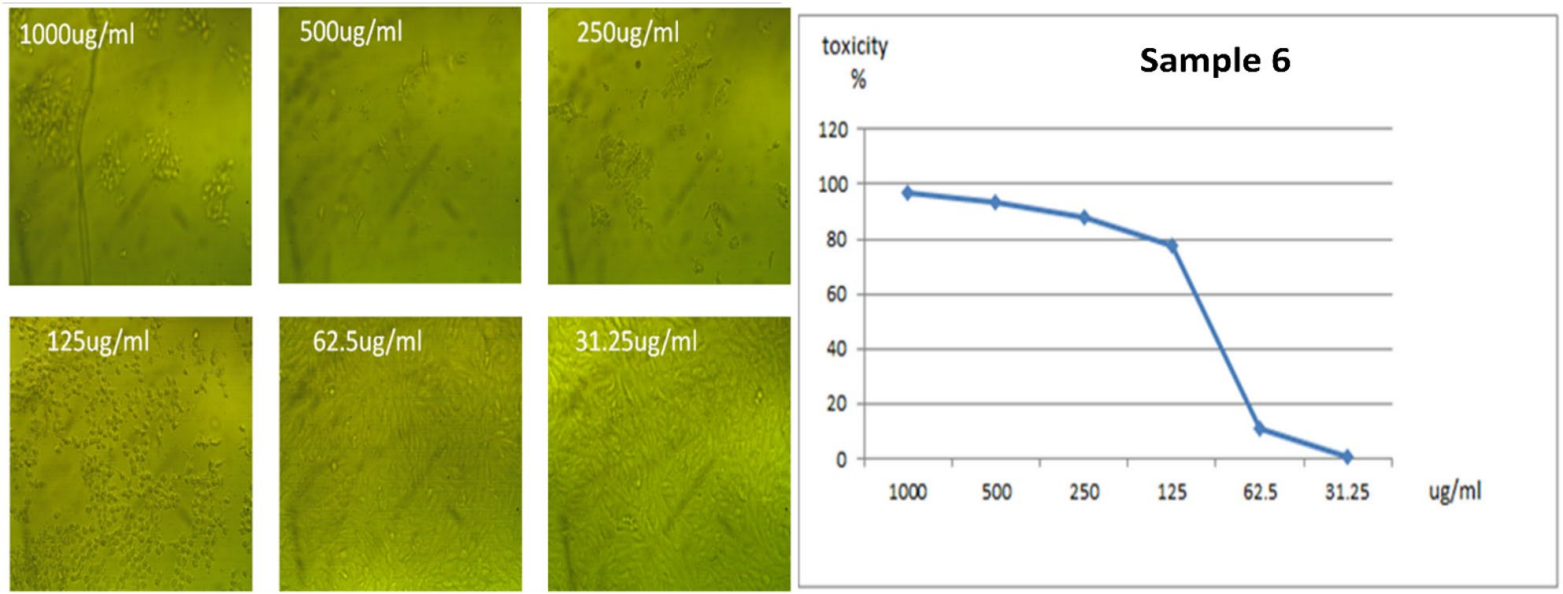
Effect of compound **6** on Vero cell at different concentration.

All the new molecules have been evaluated against normal cell lines (Vero cells), Fig. 8, which were obtained from American Type Culture Collection (ATCC, USA). The data presented in Table 5 indicate that compound **3** is lowest IC_50_ value 57.08 μg/ml and compound **4** is the higher IC_50_ ratio 119.98 μg/ml as shown in Figs. 9 and 10. Also, compound **5** and **6** had IC_50_ 90.88 μg/ml and 96.55 μg/ml respectively (Figs. 11, 12).

**Table 5:** Determination of IC_50_ of compounds **3–6** on Vero cell.

Compounds	IC50 (μg)
4	119.98
3	57.08
6	96.55
5	90.88

## Experimental

All commercially available reagents were purchased from Merck, Aldrich and Fluka and were used without further purification. All reactions were monitored by thin layer chromatography (TLC) using precoated plates of silica gel G/UV-254 of 0.25 mm thickness (Merck 60F254) using UV light (254 nm/365 nm) for visualization. Melting points were detected with a Kofler melting points apparatus and uncorrected. Infrared spectra were recorded with a FT-IR-ALPHBROKER-Platinum-ATR spectrometer and are given as cm^−1^ using the attenuated total reflection (ATR) method. ^1^H NMR and ^13^C NMR spectra for all compounds were recorded in DMSO-*d*_6_ on a Bruker Bio Spin AG spectrometer at 400 MHz and 100 MHz, respectively. Elemental analyses were obtained on a Perkin-Elmer CHN-analyzer model.

### General procedure for synthesis of compounds 3 and 4

An equimolar amount of benzil **1** (10 mmol, 2.1 g) and cyanoguanidine **2** (10 mmol, 0.84 gm) in sodium ethoxide solution (40 mol) was refluxed for 1 h. The reaction mixture filtered off and the precipitate washing several time by ethanol to give (3a,6a-diphenyltetrahydroimidazo[4,5-*d*]imidazole-2,5(1*H*,3*H*)-diylidene)dicyanamide** (4)**. After cooling, the filtrate was poured into 50 ml dist. water and acidified with hydrochloric acid; the formed product was filtered off, washed with distilled water, dried and crystallized from ethanol to give [5-oxo-4,4-diphenylimidazolidin-2-ylidene]cyanamide **(3)**.

#### [5-Oxo-4,4-diphenylimidazolidin-2-ylidene]cyanamide (3)

Yield 80%; white solid; mp: 260–262 °C; IR (ATR) ν_max_ 3567, 3403 (2N–H), 3108, 3004 (C–H arom.), 2191 (C≡N), 1765 (C=O), 1642 (C=N) cm^−1^. ^1^H NMR *δ* 7.40–7.42 (m, 10H, CH_arom._), 10.86 (s, 1H, NH), 12.26 (br. s, 1H, NH). ^13^C NMR *δ* 72.6, 115.5, 127.3, 129.0, 129.1, 138.8, 161.0, 174.9; Dept-135 NMR* δ* 127.3, 129.0, 129.1. Anal. Calcd. for C_16_H_12_N_4_O.H_2_O (294.30): C, 65.30; H, 4.79; N, 19.04. Found: C, 65.62; H, 4.53; N, 19.25.

#### (3a,6a-Diphenyltetrahydroimidazo[4,5-d]imidazole-2,5(1H,3H)-diylidene)dicyanamide (4)

Yield 5%, white solid; mp: dec. > 320 °C; IR (ATR) ν_max_ 3086 (N–H), 3032 (C–H arom.), 2204 (C≡N), 1658, 1609 (C=N) cm^−1^; ^1^H NMR *δ* 7.02–7.12 (m, 10H, CH_arom._), 9.59 (s, 4H, 4NH); ^13^C NMR *δ* 87.5, 117.3, 127.4, 128.1, 129.1, 135.5, 163.5. Anal. Calcd. for C_18_H_14_N_8_ (342.35): C, 63.15; H, 4.12; N, 32.73. Found: C, 63.46; H, 3.93; N, 32.85.

### General procedure for the synthesis of Mannich bases 5 and 6

A mixture of cyanamide **3** (1 mmol, 0.27 g) and formaldehyde solution 27% (1.1 mmol, 0.12 ml) in 40 ml refluxing absolute ethanol was stirred for 15 min and then an appropriate secondary amine; piperidine and/or morpholine (1 mmol) was added and refluxed for about 2 h (monitored with TLC). After completion of the reaction, the reaction mixture was cooled and the formed crystal **5** and/or **6**, respectively was filtered off and used without further purification.

#### [4,4-Diphenyl-5-oxo-1-(piperidin-1-ylmethyl)imidazolidin-2-ylidene]cyanamide (5)

Yield 86%; white solid; mp: 212–214 °C; IR (ATR) ν_max_ 3092 (N–H), 3001 (C–H arom.), 2934, 2847, 2810 (C–H aliph.), 2194 (C≡N), 1760 (C=O), 1627 (C=N) cm^−1^; ^1^H NMR *δ* 1.26 (s, 2H, CH_2_), 1.42 (s, 4H, 2CH_2_), 2.46 (s, 4H, 2CH_2_-N), 4.47 (s, 2H, CH_2_), 7.36–7.46 (m, 10H, CH_arom._), 11.12 (br. s, 1H, NH); ^13^C NMR *δ* 23.8, 25.9, 51.9, 63.1, 72.0, 115.4, 127.4, 129.1 (2C), 138.5, 161.7, 175.1; Dept-135 NMR *δ* 23.8 (exchangeable), 25.9 (exchangeable), 51.9 (exchangeable), 63.1 (exchangeable), 127.4, 129.0, 129.1. Anal. Calcd. for C_22_H_23_N_5_O (373.45): C, 70.76; H, 6.21; N, 18.75. Found: C, 70.98; H, 6.03; N, 18.59.

#### [4,4-Diphenyl-5-oxo-1-(morpholin-4-ylmethyl)imidazolidin-2-ylidene]cyanamide (6)

Yield 82%; white solid; mp: 209–211 °C; IR (ATR) ν_max_ 3114 (N–H), 3023 (C–H arom.), 2949, 2869, 2819 (C–H aliph.), 2188 (C≡N), 1763 (C=O), 1630 (C=N) cm^−1^; ^1^H NMR *δ* 2.49 (s, 4H, 2CH_2_-N), 3.51 (s, 4H, 2CH_2_-O), 4.50 (s, 2H, CH_2_), 7.38–7.47 (m, 10H, CH_arom._), 11.23 (br. s, 1H, NH); ^13^C NMR *δ* 51.1, 62.3, 66.5, 71.9, 115.1, 127.4, 129.1 (2C), 138.6, 161.1, 174.7. Anal. Calcd. for C_21_H_21_N_5_O_2_ (375.42): C, 67.18; H, 5.64; N, 18.65. Found: C, 67.50; H, 5.27; N, 18.48.

## X-ray crystallography

Crystals from the studied compounds were carefully examined under an optical microscope in order to choose single crystals that were appropriate and free of imperfections like fractures and bubbles. The data gathered by an Enraf–Nonius 590 diffractometer with a Kappa CCD detector and a Mo-X-ray source at room temperature^43^. The measurements were performed at the X-ray Crystallography Laboratory of the National Research Centre.

### Data analysis

The structures were solved and refined using the direct method with *SHELXS97* and *SIR92* software^44,45^ implemented in *maXus* program suit^46^. The non-hydrogen atoms were refined with anisotropic displacement parameters^47^. The general-purpose crystallographic tool *PLATON*^48^ was used for the structure analysis and presentation of the results. The molecular graphics were done using *Mercury program*^49^.

Full crystallographic included in the supplementary (Tables S1–S3), also the structures deposited at Cambridge Crystallographic Data Centre with CCDC, deposition numbers 2207479 and 1881221 for bases 3 and 6, respectively^50^.

## Antimicrobial activity

The antimicrobial activity of the tested compounds was determined by means of the agar diffusion method on Muller Hinton agar. The wells (8 mm diameter) were cut using a sterile cork borer on Muller Hinton agar (MHA, India) for bacteria and potato dextrose agar (India) for fungi. Twenty-four hours young culture of *Staphylococcus aureus* ATCC 6538, *Bacillus cereus* ATCC 10987, *Escherichia coli* ATCC 8739, *Salmonella typhimurium* ATCC 14028 and 48 h young culture of *Candida albicans* ATCC 10231 and *Aspergillus brasiliensis* ATCC 16404 were swabbed with a sterilized cotton swab on the surface of prepared Muller Hinton agar for bacteria and potato dextrose agar for fungi. One hundred microliters of dissolved compounds were loaded into each well and left for 2 h at 4 °C until the metabolite was diffused. Then the plates were incubated for 24 h at 37 °C for bacteria and 72 h at 28 °C for fungi. After incubation, the zone of inhibitions was measured and recorded^51,52^.

### In vitro cytotoxicity assaying of the prepared compounds

The prepared compounds were screened for their anti-proliferative effect against normal Vero cells. Briefly, Vero cells were seeded at a density of 104 cells per well in a sterile 96-well microplate for 24 h in a 5% CO_2_ incubator. In triplicate, different concentrations (31.25, 62.5,125, 250, 500 and 1000 µg/ml) of each prepared compound were applied to the cells and incubated for another 72 h. After discarding the medium containing compound, each well was filled with 200 ml MTT. The assay is primarily based on a biochemical reaction in which mitochondrial succinate dehydrogenase in viable cells converts the yellow tetrazolium bromide (MTT) to a purple formazan derivative. In general, the cells were cultured in RPMI-1640 medium with 10% fetal bovine serum. Penicillin (100 units/ml) and streptomycin (100 µg/ml) were added in a 5% CO2 incubator at 37 °C. Seeding of the cells was in a 96-well plate at 37 °C for 48 h under 5% CO2 and it was at a density of 1.0 × 10^4^ cells/well. After the initial incubation, the cells were treated with the new derivatives at different concentrations and incubated for 24 h. Then we add 20 µl of MTT in a solution of 5 mg/ml concentration and incubate for 4 h. To dissolve the obtained purple formazan, 100 µl of DMSO was added into each well. Measuring and recording the colorimetric assay were carried out by a plate reader (EXL 800, USA) at an absorbance of 570 nm. The percentage of relative cell viability was calculated^53^.

### Supplementary Information

Supplementary Information 1.

Supplementary Information 2.

Supplementary Information 3.

## Data Availability

The full crystallographic detailed included in Supplementary Files (Compound3-CIF, Compound6-CIF, Tables S1–S3). Also, can be obtained free of charge using deposit numbers 2207479 and 1881221, from the Cambridge Crystallographic Data Centre, 12 Union Road, Cambridge CB2 1EZ, UK; fax: (+44) 1223-336-033; or e-mail: deposit@ccdc.cam.ac.uk.
