# Supplementary material for: Synthesis, X-ray crystallography and antimicrobial activity of 2-cyanoguanidinophenytoin
Source: Sci Rep. 2023 Nov 9;13:19510. doi: 10.1038/s41598-023-45533-1 (PMC10636160; doi:10.1038/s41598-023-45533-1)
Supplement: Supplementary file 3 — Supplementary Information 3. [file 41598_2023_45533_MOESM3_ESM.docx]

Supporting Information

**Synthesis, X-ray crystallography and antimicrobial activity of 2-Cyanoguanidinophenytoin**

Ahmed F. Mabieda**, Amr H. Moustafab,c*, Antar A. Abdelhamidb, Taha M. Tiamad, Amer A. Amerb

*aX-Ray Crystallography Lab., Solid State Physics Department, National Research Centre, Dokki 12622, Giza, Egypt.*

*bDepartment of Chemistry, Faculty of Science, Sohag University, Sohag 82524, Egypt;*

*cFaculty of Science, King Salman International University, Ras sudr, Sinai 46612, Egypt;*

*dDepartment of Basic Sciences, October High Institute of Engineering & Technology - OHI, 6th of October City, Giza, Egypt;*

*E-mail: [amr_hassanegypt@ymail.com](mailto:amr_hassanegypt@ymail.com), Fax: +20934601159, Mobil: +201028052886, Mobil: +201028052886

**E-mail: [af.mabied@nrc.sci.eg](mailto:af.mabied@nrc.sci.eg)

**Contents**

[General Information 2](#_Toc142304824)

[1. General Information: 2](#_Toc142304825)

[2. Optimized condition for the synthesis 2](#_Toc142304826)

[3. General procedure for the synthesis 3](#_Toc142304827)

[NMR Spectra 5](#_Toc142304828)

[1H, 13C and Dept-135 NMR Spectra of 3: 5](#_Toc142304829)

[1H and 13C NMR Spectra of 4: 8](#_Toc142304830)

[1H, 13C and Dept-135 NMR Spectra of 5: 10](#_Toc142304831)

[1H and 13C NMR Spectra of 6: 14](#_Toc142304832)

[X-ray Crystallography 17](#_Toc142304833)

[In vitro cytotoxicity 21](#_Toc142304834)

# General Information

## 1. General Information:

All commercially available reagents were purchased from Merck, Aldrich and Fluka and were used without further purification. All reactions were monitored by thin layer chromatography (TLC) using precoated plates of silica gel G/UV-254 of 0.25 mm thickness (Merck 60F254) using UV light (254 nm/365 nm) for visualization. Melting points were detected with a Kofler melting points apparatus and uncorrected. Infrared spectra were recorded with a FT-IR-ALPHBROKER-Platinum-ATR spectrometer and are given as cm-1 using the attenuated total reflection (ATR) method. 1H NMR and 13C NMR spectra for all compounds were recorded in DMSO-*d6* on a Bruker Bio Spin AG spectrometer at 400 MHz and 100 MHz, respectively. For 1H NMR, chemical shifts (δ) were given in parts per million (ppm) with reference to tetramethylsilane (TMS) as an internal standard (δ=0); coupling constants (J) were given in hertz (Hz) and data are reported as follows: chemical shift, integration, multiplicity (s = singlet, m = multiplet). Elemental analyses were obtained on a Perkin-Elmer CHN-analyzer model.

## 2. Optimized condition for the synthesis

**[5-oxo-4,4-diphenylimidazolidin-2-ylidene]cyanamide (3)** **and** **(3a,6a-Diphenyltetrahydroimidazo[4,5-*d*]imidazole-2,5(1*H*,3*H*)-diylidene)dicyanamide (4)**

An equimolar amount of benzil **1** (10 mmol, 2.1 gm) and cyanoguanidine **2** (10 mmol, 0.84 gm) in sodium ethoxide solution (40 mol) was refluxed for 1 hr. The reaction mixture filtered off and the precipitate watching several time by ethanol to give (3a,6a-Diphenyltetrahydroimidazo[4,5-*d*]imidazole-2,5(1*H*,3*H*)-diylidene)dicyanamide **(4)**. After cooling, the filtrate was poured into 50 mL dist. water and acidified with hydrochloric acid; the formed product was filtered off, washed with distilled water, dried and crystallized from ethanol to give [5-oxo-4,4-diphenylimidazolidin-2-ylidene]cyanamide **(3)**.

**[5-oxo-4,4-diphenylimidazolidin-2-ylidene]cyanamide (3)**

Yield 80 %; white solid; mp: 260–262 °C; IR (ATR) max 3567, 3403 (2N–H), 3108, 3004 (C–H arom.), 2191 (C≡N), 1765 (C=O), 1642 (C=N) cm-1. 1HNMR *δ* 7.40-7.42 (m, 10H, CHarom.), 10.86 (s, 1H, NH), 12.26 (br. s, 1H, NH). 13CNMR *δ* 72.6, 115.5, 127.3, 129.0, 129.1, 138.8, 161.0, 174.9; Dept-135 NMR *δ* 127.3, 129.0, 129.1. Anal. Calcd. for C16H12N4O.H2O (294.30): C, 65.30; H, 4.79; N, 19.04. Found: C, 65.62; H, 4.53; N, 19.25.

**(3a,6a-Diphenyltetrahydroimidazo[4,5-*d*]imidazole-2,5(1*H*,3*H*)-diylidene)dicyanamide (4)**

Yield 5%, white solid; mp: dec. > 320 °C; IR (ATR) max 3086 (N–H), 3032 (C–H arom.), 2204 (C≡N), 1658, 1609 (C=N) cm-1; 1H NMR *δ* 7.02-7.12 (m, 10H, CHarom.), 9.59 (s, 4H, 4NH); 13C NMR *δ* 87.5, 117.3, 127.4, 128.1, 129.1, 135.5, 163.5. Anal. Calcd. for C18H14N8 (342.35): C, 63.15; H, 4.12; N, 32.73. Found: C, 63.46; H, 3.93; N, 32.85.

## 3. General procedure for the synthesis

**Mannich bases 5 and 6:**

A mixture of cyanamide **3** (1 mmol, 0.27 gm) and formaldehyde solution 27 % (1.1 mmol, 0.12 mL) in 40 mL refluxing absolute ethanol was stirred for 15 mins and then an appropriate secondary amine; piperidine and/or morpholine (1 mmol) was added and refluxed for about 2 hrs (monitored with TLC). After completion of the reaction, the reaction mixture was cooled and the formed crystal **5** and/or **6**, respectively was filtered off and used without further purification.

**[4,4-Diphenyl-5-oxo-1-(piperidin-1-ylmethyl)imidazolidin-2-ylidene]cyanamide (5)**

Yield 86 %; white solid; mp: 212-214 °C; IR (ATR) max 3092 (N–H), 3001 (C–H arom.), 2934, 2847, 2810 (C–H aliph.), 2194 (C≡N), 1760 (C=O), 1627 (C=N) cm-1; 1H NMR *δ* 1.26 (s, 2H, CH2), 1.42 (s, 4H, 2CH2), 2.46 (s, 4H, 2CH2-N), 4.47 (s, 2H, CH2), 7.36–7.46 (m, 10H, CHarom.), 11.12 (br. s, 1H, NH); 13C NMR *δ* 23.8, 25.9, 51.9, 63.1, 72.0, 115.4, 127.4, 129.1 (2C), 138.5, 161.7, 175.1; Dept-135 NMR *δ* 23.8 (exchangeable), 25.9 (exchangeable), 51.9 (exchangeable), 63.1 (exchangeable), 127.4, 129.0, 129.1. Anal. Calcd. for C22H23N5O (373.45): C, 70.76; H, 6.21; N, 18.75. Found: C, 70.98; H, 6.03; N, 18.59.

**[4,4-Diphenyl-5-oxo-1-(morpholin-4-ylmethyl)imidazolidin-2-ylidene]cyanamide (6)**

Yield 82 %; white solid; mp: 209-211 °C; IR (ATR) max 3114 (N–H), 3023 (C–H arom.), 2949, 2869, 2819 (C–H aliph.), 2188 (C≡N), 1763 (C=O), 1630 (C=N) cm-1; 1H NMR *δ* 2.49 (s, 4H, 2CH2-N), 3.51 (s, 4H, 2CH2-O), 4.50 (s, 2H, CH2), 7.38–7.47 (m, 10H, CHarom.), 11.23 (br. s, 1H, NH); 13C NMR *δ* 51.1, 62.3, 66.5, 71.9, 115.1, 127.4, 129.1 (2C), 138.6, 161.1, 174.7. Anal. Calcd. for C21H21N5O2 (375.42): C, 67.18; H, 5.64; N, 18.65. Found: C, 67.50; H, 5.27; N, 18.48.

# NMR Spectra

## **1H, 13C and Dept-135 NMR Spectra of 3**:

**
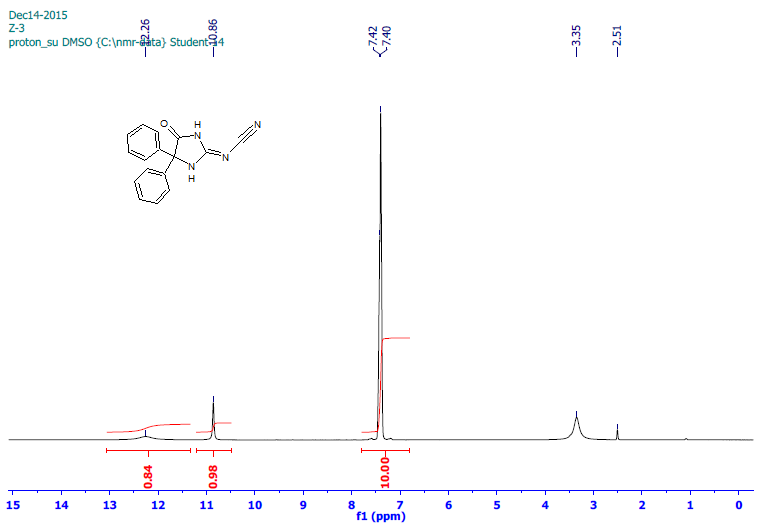
**

**
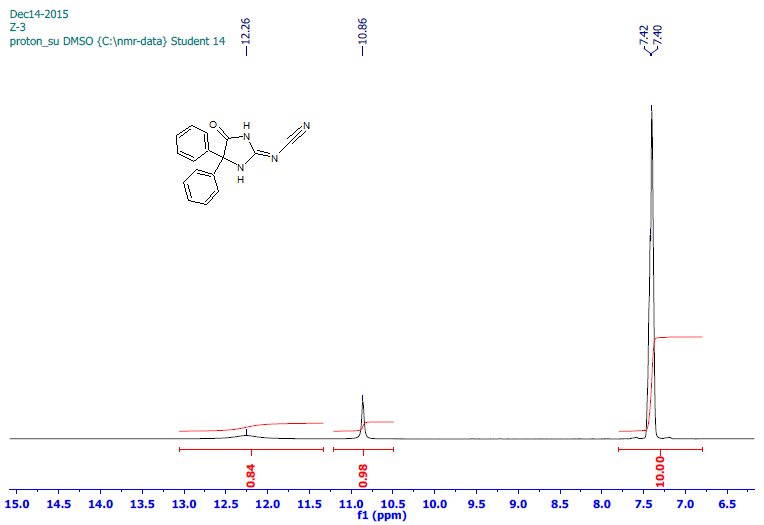
**

**
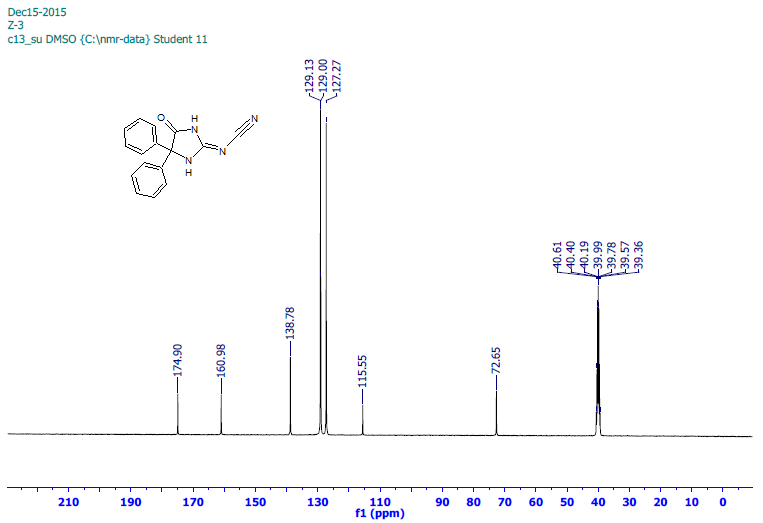
**

**
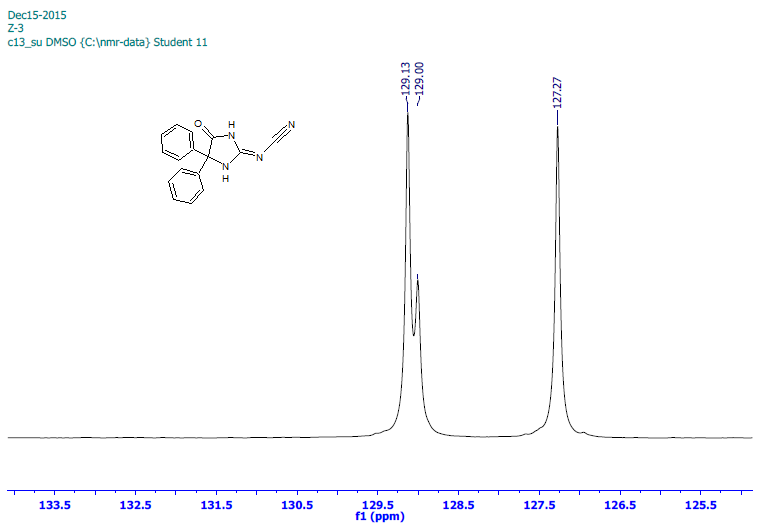
**

**
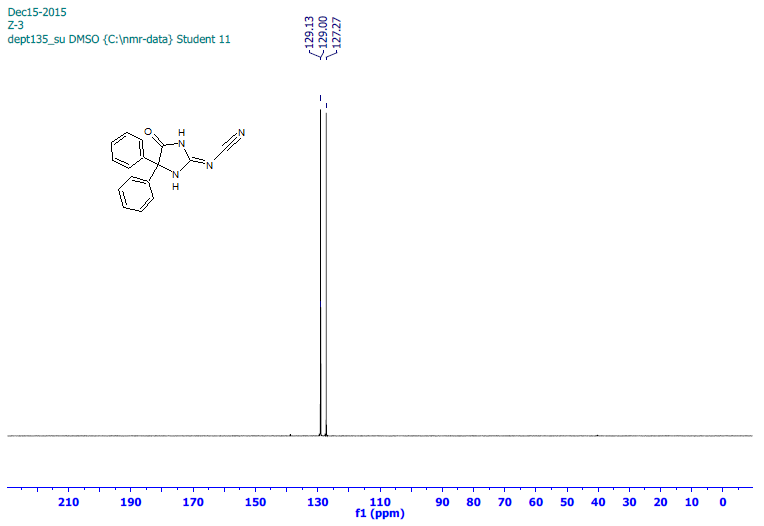
**

**
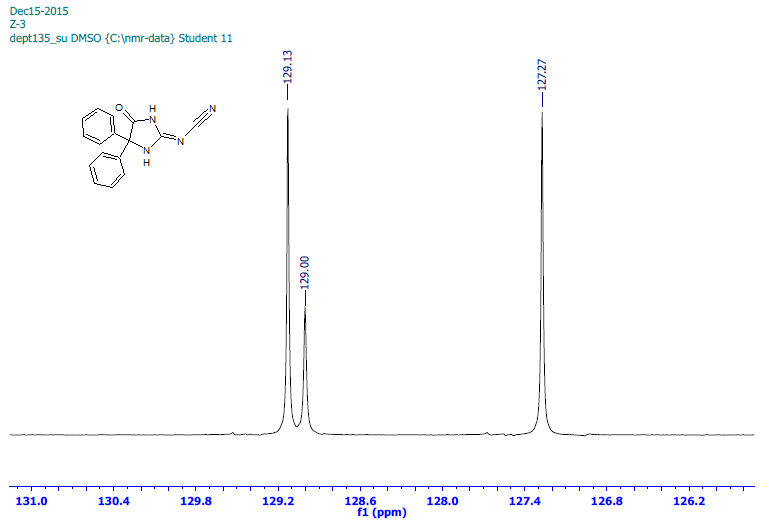
**

## **1H and 13C NMR Spectra of 4**:

**
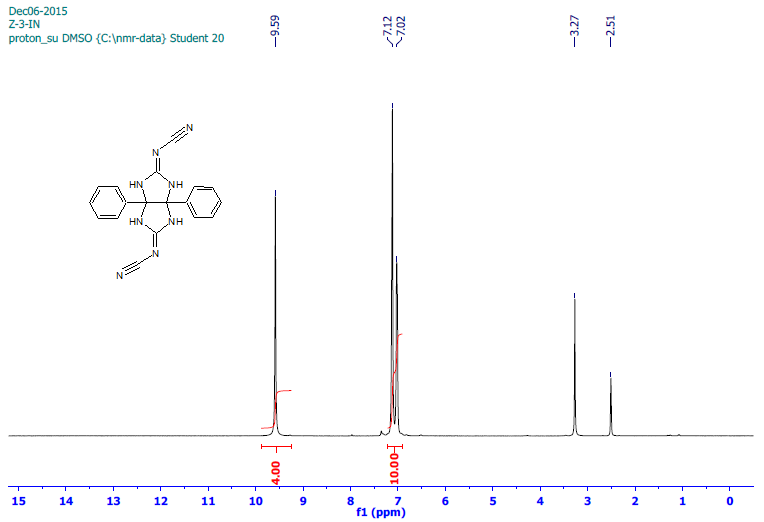
**

**
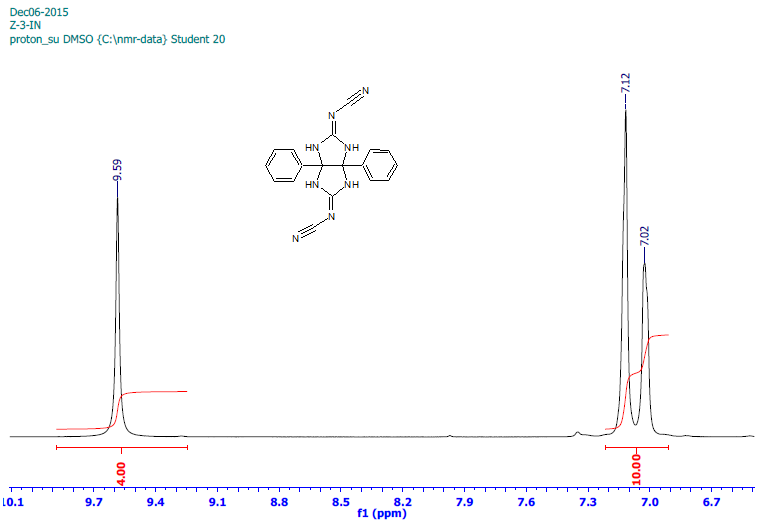
**

**
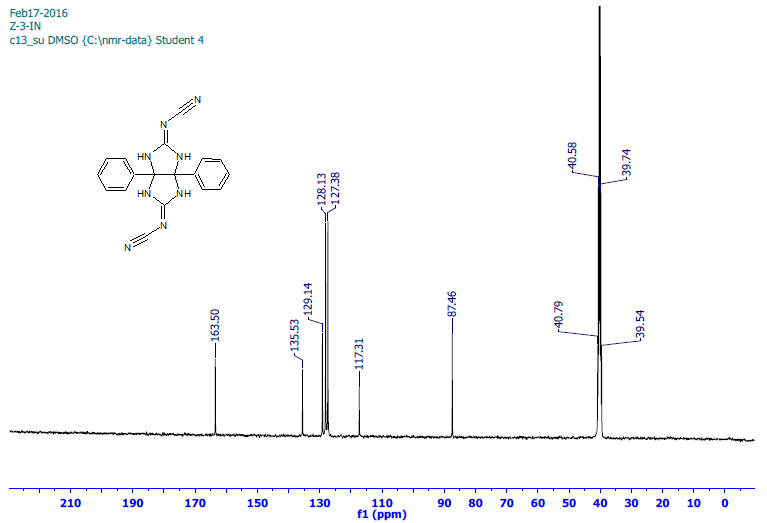
**

**
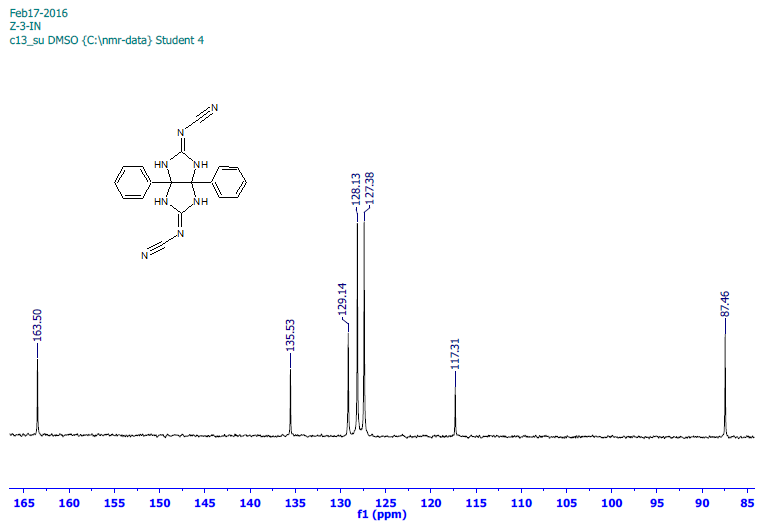
**

## **1H, 13C and Dept-135 NMR Spectra of 5**:

**
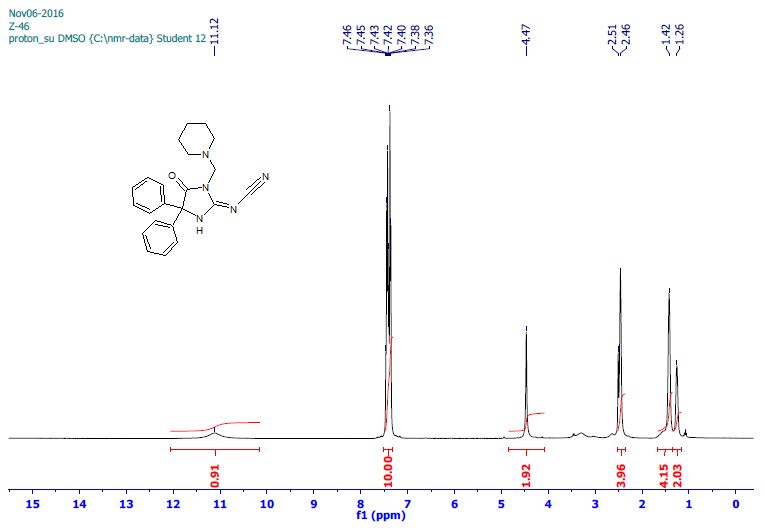
**

**
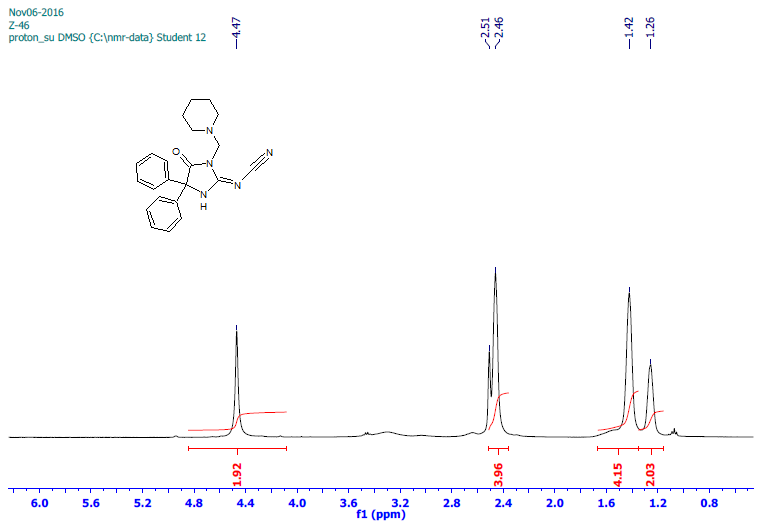
**

**
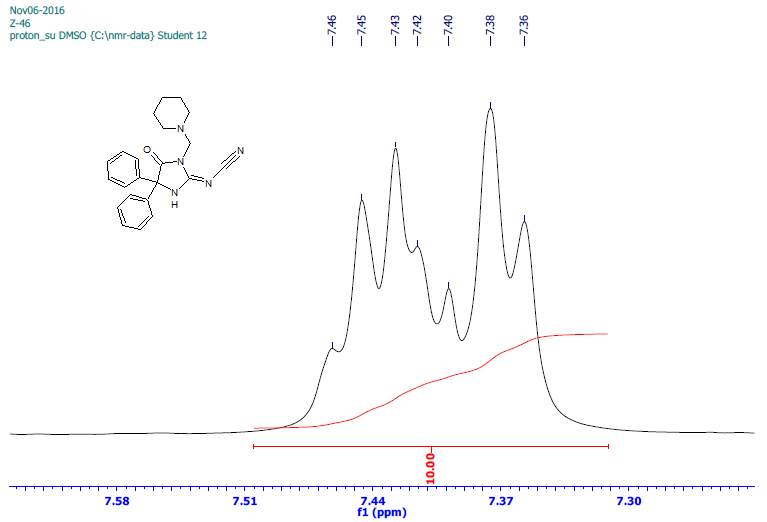
**

**
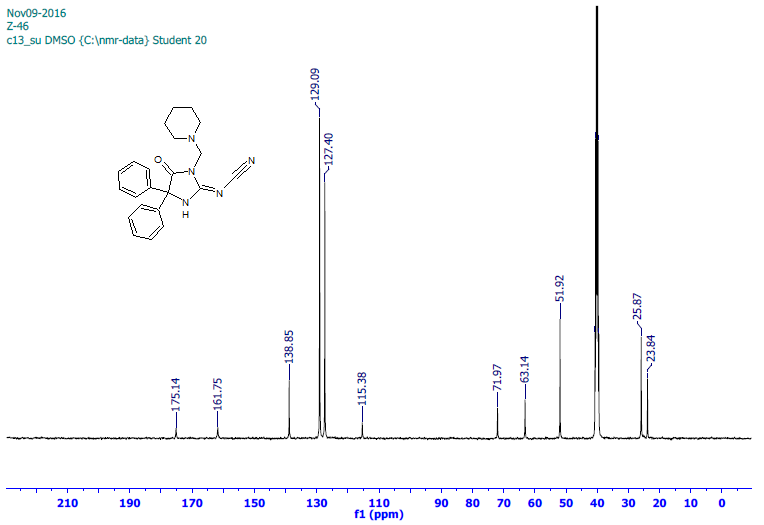
**

**
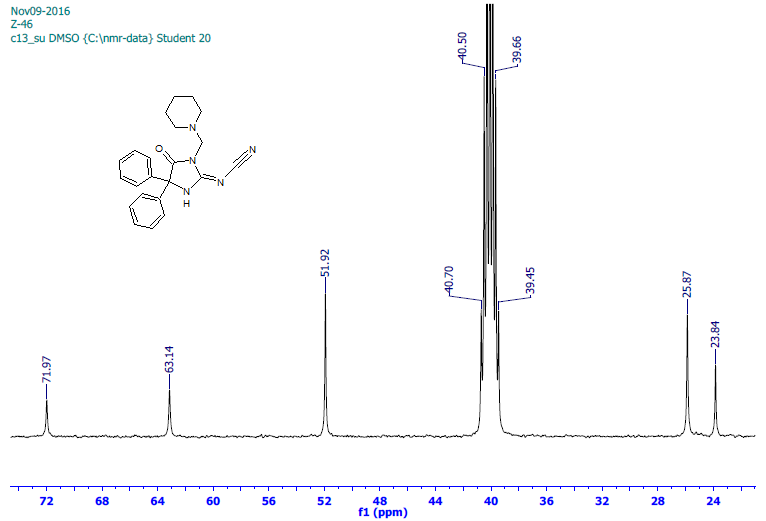
**

**
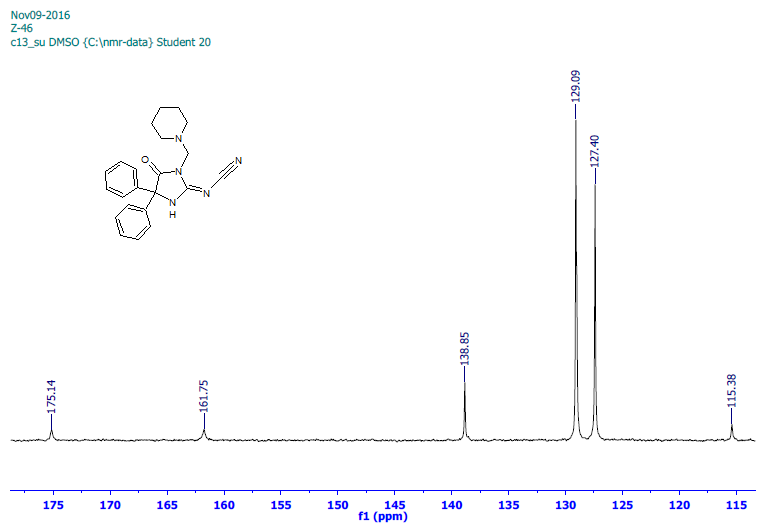
**

**
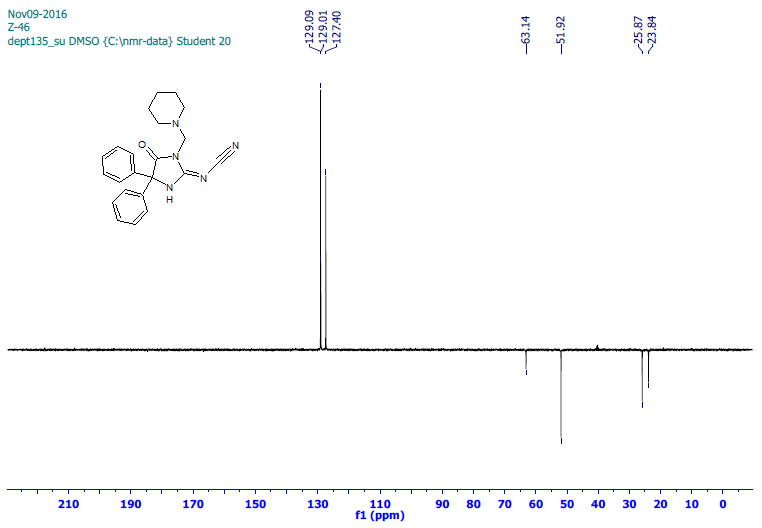
**

**
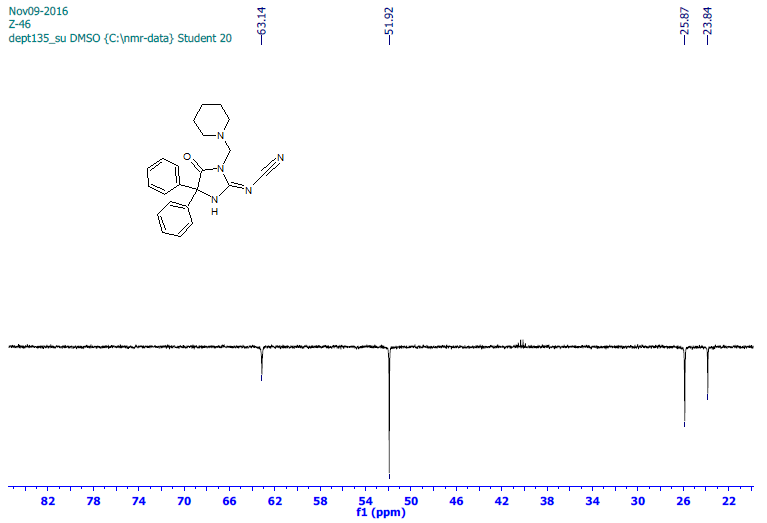
**

**
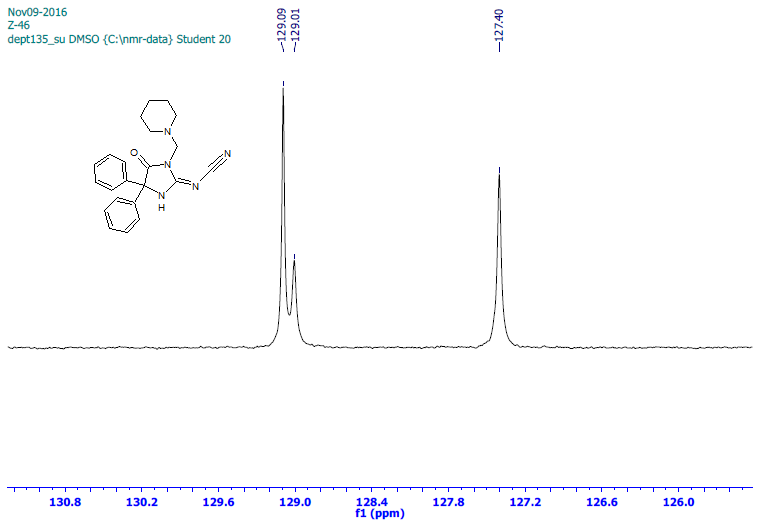
**

## **1H and 13C NMR Spectra of 6**:

**
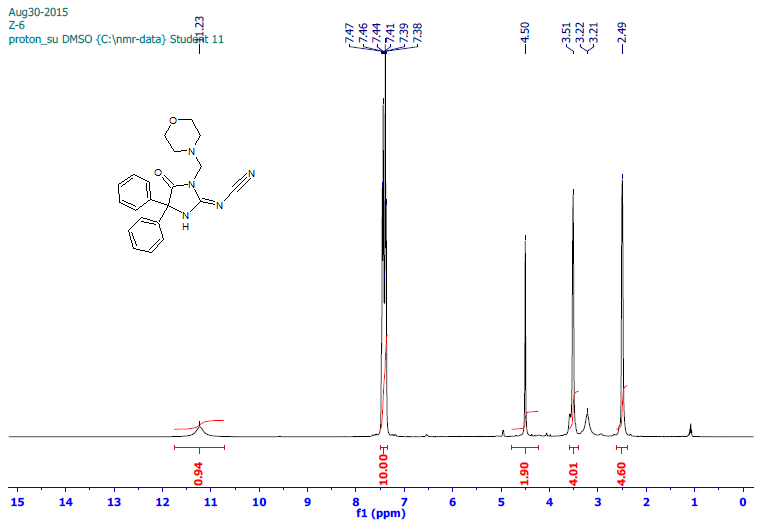
**

**
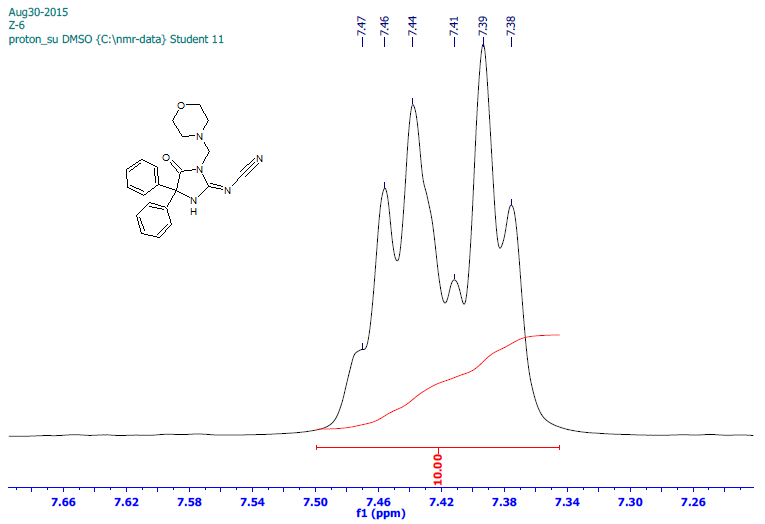
**

**
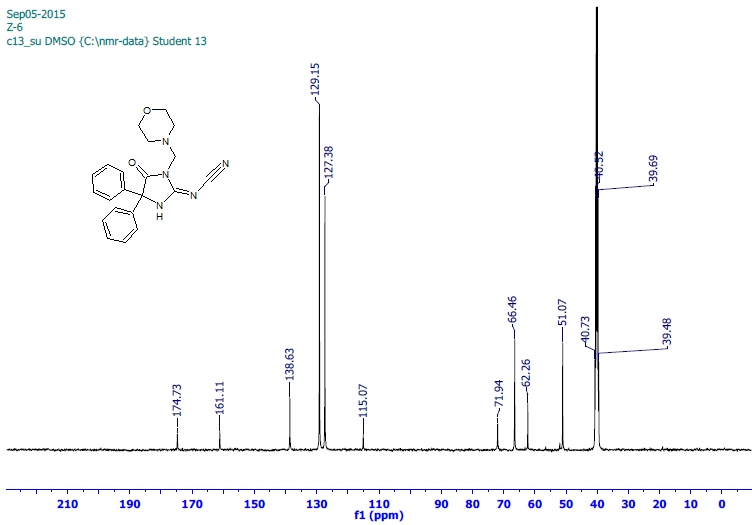
**

**
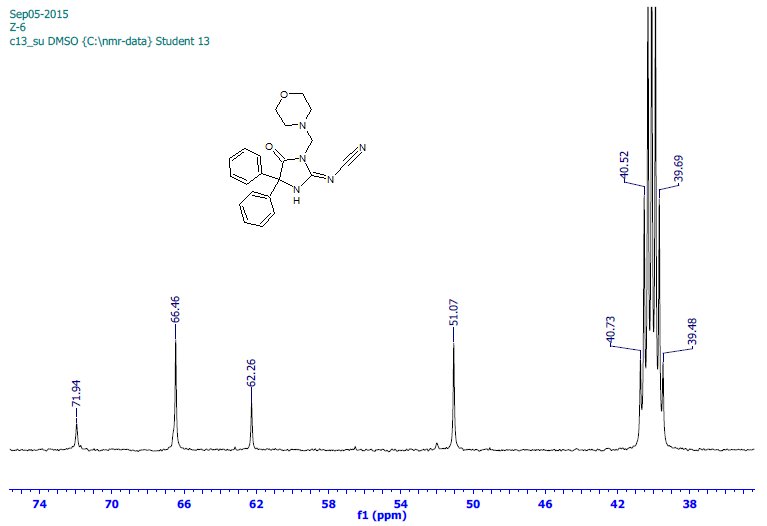
**

**
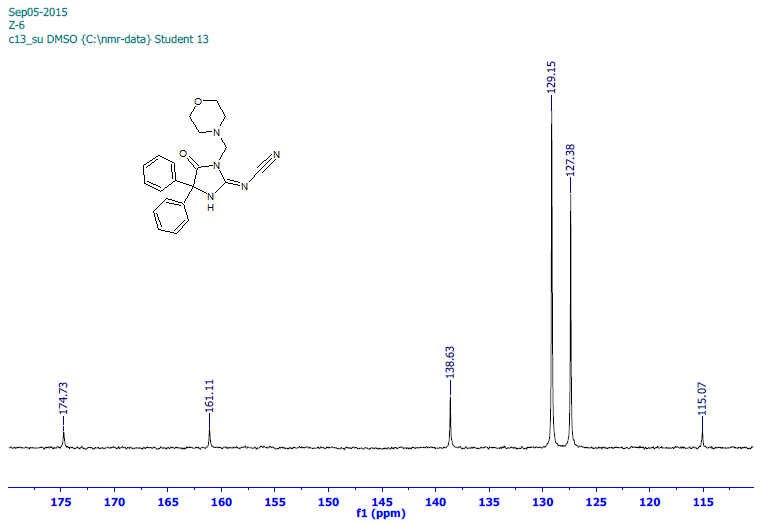
**

# X-ray Crystallography

**Table S1. Experimental details**

Experiments were carried out at 298 K with Mo *K*α radiation using a KappaCCD. Only H-atom coordinates were refined.

|  | 3 | 6 |
| --- | --- | --- |
| Crystal data | | |
| Deposition Number  CCDC | 2207479 | 1881221 |
| Chemical formula | C16H12N4O·H2O | C21H21N5O2 |
| *M*r | 294.31 | 375.43 |
| Crystal system, space group | Monoclinic, *P*21/*c* | Monoclinic, *C*2/*c* |
| *a*, *b*, *c* (Å) | 11.5919 (5), 10.6957 (4), 11.5810 (4) | 13.7522 (4), 10.6925 (3), 27.1106 (10) |
| β (°) | 101.4697 (14) | 93.2292 (10) |
| *V* (Å3) | 1407.18 (9) | 3980.2 (2) |
| *Z* | 4 | 8 |
|  (mm-1) | ? | ? |
| Crystal size (mm) | 0.19 × 0.18 × 0.15 | 0.19 × 0.17 × 0.15 |
|  | | |
| Data collection | | |
| Absorption correction | – | Multi-scan  multi-scan from symmetry-related measurements *SORTAV* (Blessing 1995) |
| *T*min, *T*max | – | 0.925, 0.986 |
| No. of measured, independent and  observed [*I* > 2σ(*I*)] reflections | 18967, 4059, 1640 | 5594, 5328, 1017 |
| *R*int | 0.12 | 0.105 |
| (sin /λ)max (Å-1) | 0.705 | 0.704 |
|  | | |
| Refinement | | |
| *R*[*F*2 > 2σ(*F*2)], *wR*(*F*2), *S* | 0.067, 0.208, 0.94 | 0.046, 0.200, 0.6 |
| No. of reflections | 4059 | 5328 |
| No. of parameters | 199 | 253 |
| max, min (e Å-3) | 0.32, -0.45 | 0.13, -0.15 |

Computer programs: KappaCCD, *HKL* *SCALEPACK* (Otwinowski & Minor 1997), *DENZO* and Scalepak (Otwinowski & Minor, 1997), *SIR92* (Altomare *et al.*, 1994), *SHELXL97* (Sheldrick, 1997).

**Table S2. Selected geometric parameters (Å, º) of 3**

| O1—C7 | 1.218 (3) | C10—C18 | 1.387 (4) |
| --- | --- | --- | --- |
| N3—C7 | 1.360 (3) | C11—C16 | 1.380 (3) |
| N3—C9 | 1.363 (3) | C11—C20 | 1.377 (4) |
| N4—C9 | 1.318 (3) | C12—C14 | 1.396 (4) |
| N4—C5 | 1.467 (3) | C14—C15 | 1.382 (4) |
| C5—C12 | 1.527 (3) | C15—C22 | 1.369 (4) |
| C5—C7 | 1.538 (4) | C16—C17 | 1.386 (3) |
| C5—C11 | 1.542 (3) | C17—C19 | 1.362 (4) |
| N6—C9 | 1.324 (3) | C18—C22 | 1.372 (4) |
| N6—C13 | 1.326 (3) | C19—C21 | 1.373 (4) |
| N8—C13 | 1.153 (3) | C20—C21 | 1.388 (4) |
| C10—C12 | 1.391 (3) |  |  |
|  | | | |
| C7—N3—C9 | 111.1 (2) | C16—C11—C20 | 118.5 (2) |
| C9—N4—C5 | 112.2 (2) | C16—C11—C5 | 119.1 (2) |
| N4—C5—C12 | 111.76 (19) | C20—C11—C5 | 122.3 (2) |
| N4—C5—C7 | 99.73 (18) | C10—C12—C14 | 117.9 (2) |
| C12—C5—C7 | 109.62 (18) | C10—C12—C5 | 122.0 (2) |
| N4—C5—C11 | 111.22 (18) | C14—C12—C5 | 120.1 (2) |
| C12—C5—C11 | 111.83 (19) | N8—C13—N6 | 173.7 (3) |
| C7—C5—C11 | 112.11 (19) | C15—C14—C12 | 120.6 (3) |
| C9—N6—C13 | 118.6 (2) | C22—C15—C14 | 120.8 (3) |
| O1—C7—N3 | 125.4 (2) | C11—C16—C17 | 120.5 (2) |
| O1—C7—C5 | 127.4 (2) | C19—C17—C16 | 120.7 (3) |
| N3—C7—C5 | 107.1 (2) | C22—C18—C10 | 120.6 (3) |
| N4—C9—N6 | 130.4 (2) | C17—C19—C21 | 119.4 (2) |
| N4—C9—N3 | 109.7 (2) | C11—C20—C21 | 120.6 (3) |
| N6—C9—N3 | 119.9 (2) | C19—C21—C20 | 120.2 (3) |
| C12—C10—C18 | 120.6 (3) | C15—C22—C18 | 119.4 (3) |

**Table S3. Selected geometric parameters (Å, º) of 6**

| O1—C9 | 1.203 (4) | C20—C23 | 1.380 (6) |
| --- | --- | --- | --- |
| N2—C11 | 1.315 (4) | C21—C28 | 1.363 (6) |
| N2—C8 | 1.335 (5) | C23—C28 | 1.361 (6) |
| N3—C11 | 1.329 (4) | C24—C25 | 1.350 (5) |
| N3—C16 | 1.480 (4) | C24—C27 | 1.373 (6) |
| N4—C11 | 1.375 (4) | N3—H3 | 0.9600 |
| N4—C9 | 1.378 (4) | C10—H10A | 0.9600 |
| N4—C10 | 1.497 (4) | C10—H10B | 0.9600 |
| N5—C8 | 1.142 (4) | C14—H14A | 0.9600 |
| N6—C10 | 1.424 (4) | C14—H14B | 0.9601 |
| N6—C14 | 1.460 (4) | C15—H15 | 0.9600 |
| N6—C18 | 1.458 (4) | C17—H17 | 0.9601 |
| O7—C26 | 1.411 (4) | C18—H18A | 0.9600 |
| O7—C22 | 1.413 (4) | C18—H18B | 0.9599 |
| C9—C16 | 1.540 (5) | C19—H19 | 0.9601 |
| C12—C15 | 1.365 (5) | C20—H20 | 0.9599 |
| C12—C17 | 1.368 (5) | C21—H21 | 0.9599 |
| C12—C16 | 1.540 (5) | C22—H22A | 0.9600 |
| C13—C20 | 1.372 (5) | C22—H22B | 0.9600 |
| C13—C19 | 1.374 (5) | C23—H23 | 0.9599 |
| C13—C16 | 1.534 (5) | C24—H24 | 0.9600 |
| C14—C22 | 1.506 (5) | C25—H25 | 0.9599 |
| C15—C27 | 1.398 (5) | C26—H26A | 0.9601 |
| C17—C25 | 1.389 (5) | C26—H26B | 0.9600 |
| C18—C26 | 1.514 (5) | C27—H27 | 0.9600 |
| C19—C21 | 1.383 (6) | C28—H28 | 0.9600 |
|  | | | |
| C11—N2—C8 | 115.7 (3) | N6—C10—H10A | 106.7 |
| C11—N3—C16 | 112.7 (3) | N4—C10—H10A | 109.8 |
| C11—N4—C9 | 111.3 (3) | N6—C10—H10B | 105.7 |
| C11—N4—C10 | 123.6 (3) | N4—C10—H10B | 108.2 |
| C9—N4—C10 | 125.1 (3) | H10A—C10—H10B | 109.5 |
| C10—N6—C14 | 114.3 (3) | N6—C14—H14A | 109.1 |
| C10—N6—C18 | 114.8 (3) | C22—C14—H14A | 107.6 |
| C14—N6—C18 | 111.5 (3) | N6—C14—H14B | 109.2 |
| C26—O7—C22 | 109.1 (3) | C22—C14—H14B | 112.2 |
| N5—C8—N2 | 175.7 (4) | H14A—C14—H14B | 109.5 |
| O1—C9—N4 | 125.7 (4) | C12—C15—H15 | 118.8 |
| O1—C9—C16 | 127.3 (4) | C27—C15—H15 | 120.7 |
| N4—C9—C16 | 107.0 (3) | C12—C17—H17 | 118.7 |
| N6—C10—N4 | 116.8 (3) | C25—C17—H17 | 120.4 |
| N2—C11—N3 | 129.5 (4) | N6—C18—H18A | 111.1 |
| N2—C11—N4 | 121.5 (4) | C26—C18—H18A | 112.3 |
| N3—C11—N4 | 108.9 (3) | N6—C18—H18B | 108.4 |
| C15—C12—C17 | 119.1 (4) | C26—C18—H18B | 106.7 |
| C15—C12—C16 | 123.9 (4) | H18A—C18—H18B | 109.5 |
| C17—C12—C16 | 117.0 (4) | C13—C19—H19 | 118.5 |
| C20—C13—C19 | 118.9 (4) | C21—C19—H19 | 121.6 |
| C20—C13—C16 | 118.7 (4) | C13—C20—H20 | 118.2 |
| C19—C13—C16 | 122.4 (4) | C23—C20—H20 | 121.5 |
| N6—C14—C22 | 109.3 (3) | C28—C21—H21 | 119.9 |
| C12—C15—C27 | 120.5 (4) | C19—C21—H21 | 119.0 |
| N3—C16—C13 | 112.3 (3) | O7—C22—H22A | 107.9 |
| N3—C16—C12 | 108.8 (3) | C14—C22—H22A | 107.1 |
| C13—C16—C12 | 112.4 (3) | O7—C22—H22B | 109.2 |
| N3—C16—C9 | 99.7 (3) | C14—C22—H22B | 110.8 |
| C13—C16—C9 | 111.1 (3) | H22A—C22—H22B | 109.5 |
| C12—C16—C9 | 111.8 (3) | C28—C23—H23 | 118.6 |
| C12—C17—C25 | 120.8 (4) | C20—C23—H23 | 120.6 |
| N6—C18—C26 | 108.7 (4) | C25—C24—H24 | 119.8 |
| C13—C19—C21 | 119.9 (4) | C27—C24—H24 | 119.5 |
| C13—C20—C23 | 120.4 (4) | C24—C25—H25 | 120.5 |
| C28—C21—C19 | 121.1 (5) | C17—C25—H25 | 119.8 |
| O7—C22—C14 | 112.3 (4) | O7—C26—H26A | 108.4 |
| C28—C23—C20 | 120.8 (5) | C18—C26—H26A | 110.7 |
| C25—C24—C27 | 120.7 (4) | O7—C26—H26B | 109.7 |
| C24—C25—C17 | 119.7 (4) | C18—C26—H26B | 107.3 |
| O7—C26—C18 | 111.3 (4) | H26A—C26—H26B | 109.5 |
| C24—C27—C15 | 119.2 (4) | C24—C27—H27 | 118.9 |
| C23—C28—C21 | 118.8 (5) | C15—C27—H27 | 121.9 |
| C11—N3—H3 | 118.6 | C23—C28—H28 | 120.6 |
| C16—N3—H3 | 128.7 | C21—C28—H28 | 120.5 |

# In vitro cytotoxicity

**Table S4:** determination of IC50 of compounds **3-6** on Vero cell.

| **ID** | **ug/ml** | **O.D** | | | **Mean O.D** | **ST.E** | **Viability %** | **Toxicity %** | **IC50** |
| --- | --- | --- | --- | --- | --- | --- | --- | --- | --- |
| vero | -------- | 0.629 | 0.641 | 0.656 | 0.642 | 0.00781 | 100 | 0 | ug |
| **4** | 1000 | 0.017 | 0.021 | 0.022 | 0.02 | 0.001528 | 3.115264798 | 96.8847352 | 119.98 |
|  | 500 | 0.075 | 0.058 | 0.083 | 0.072 | 0.007371 | 11.21495327 | 88.78504673 |  |
|  | 250 | 0.143 | 0.12 | 0.122 | 0.128333 | 0.007356 | 19.98961578 | 80.01038422 |  |
|  | 125 | 0.285 | 0.217 | 0.324 | 0.275333 | 0.031264 | 42.88681205 | 57.11318795 |  |
|  | 62.5 | 0.645 | 0.637 | 0.618 | 0.633333 | 0.008007 | 98.65005192 | 1.349948079 |  |
|  | 31.25 | 0.644 | 0.629 | 0.64 | 0.637667 | 0.004485 | 99.32502596 | 0.674974039 |  |
| **3** | 1000 | 0.015 | 0.017 | 0.015 | 0.015667 | 0.000667 | 2.440290758 | 97.55970924 | 57.08 |
|  | 500 | 0.034 | 0.062 | 0.039 | 0.045 | 0.008622 | 7.009345794 | 92.99065421 |  |
|  | 250 | 0.037 | 0.059 | 0.05 | 0.048667 | 0.006386 | 7.580477674 | 92.41952233 |  |
|  | 125 | 0.163 | 0.128 | 0.135 | 0.142 | 0.010693 | 22.11838006 | 77.88161994 |  |
|  | 62.5 | 0.266 | 0.231 | 0.197 | 0.231333 | 0.019919 | 36.03322949 | 63.96677051 |  |
|  | 31.25 | 0.467 | 0.431 | 0.444 | 0.447333 | 0.010525 | 69.6780893 | 30.3219107 |  |
| **6** | 1000 | 0.019 | 0.018 | 0.02 | 0.019 | 0.000577 | 2.959501558 | 97.04049844 | 96.55 |
|  | 500 | 0.035 | 0.042 | 0.047 | 0.041333 | 0.00348 | 6.438213915 | 93.56178609 |  |
|  | 250 | 0.086 | 0.079 | 0.063 | 0.076 | 0.006807 | 11.83800623 | 88.16199377 |  |
|  | 125 | 0.164 | 0.118 | 0.148 | 0.143333 | 0.013482 | 22.32606438 | 77.67393562 |  |
|  | 62.5 | 0.593 | 0.564 | 0.558 | 0.571667 | 0.010806 | 89.04465213 | 10.95534787 |  |
|  | 31.25 | 0.652 | 0.634 | 0.629 | 0.638333 | 0.006984 | 99.42886812 | 0.57113188 |  |
| **5** | 1000 | 0.015 | 0.018 | 0.017 | 0.016667 | 0.000882 | 2.596053998 | 97.403946 | 90.88 |
|  | 500 | 0.032 | 0.057 | 0.047 | 0.045333 | 0.007265 | 7.061266874 | 92.93873313 |  |
|  | 250 | 0.122 | 0.11 | 0.145 | 0.125667 | 0.010269 | 19.57424714 | 80.42575286 |  |
|  | 125 | 0.246 | 0.189 | 0.201 | 0.212 | 0.017349 | 33.02180685 | 66.97819315 |  |
|  | 62.5 | 0.378 | 0.341 | 0.36 | 0.359667 | 0.010682 | 56.02284528 | 43.97715472 |  |
|  | 31.25 | 0.586 | 0.615 | 0.603 | 0.601333 | 0.008413 | 93.66562825 | 6.334371755 |  |
